# Supplementary material for: Regulation of BRCA1 stability through the tandem UBX domains of isoleucyl-tRNA synthetase 1
Source: Nat Commun. 2022 Nov 8;13:6732. doi: 10.1038/s41467-022-34612-y (PMC9643514; doi:10.1038/s41467-022-34612-y)
Supplement: Supplementary file 1 — Supplementary Information [file 41467_2022_34612_MOESM1_ESM.pdf]

Supplementary information

Regulation of BRCA1 stability through the tandem UBX domains of isoleucyl-tRNA synthetase 1

Supplementary Figure 1–11
Supplementary Note
Supplementary Methods
Supplementary Table 1 and 2
Supplementary References

Supplementary Figures

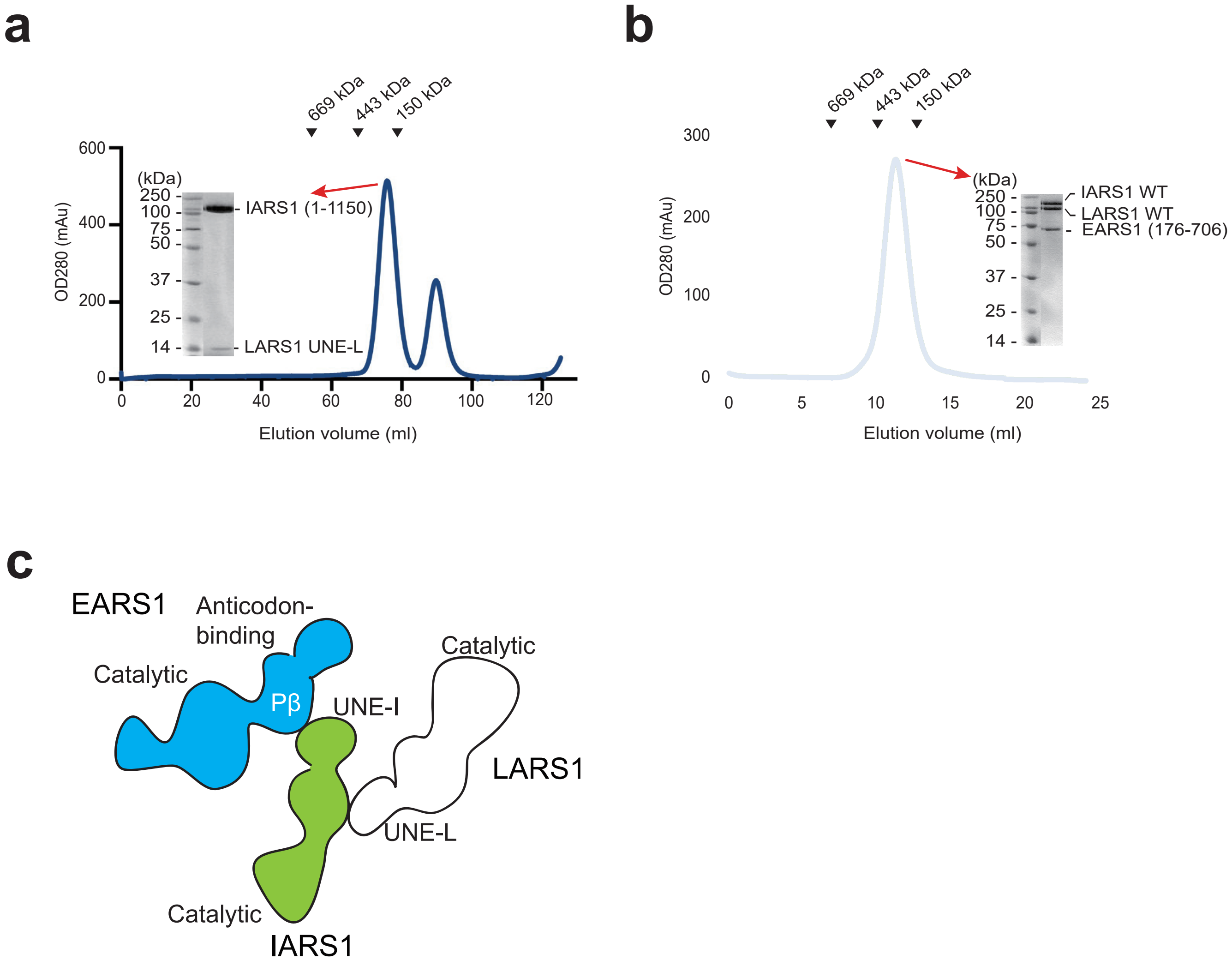

Supplementary Figure 1 EARS1, IARS1, and LARS1 form a ternary complex. a A mixture of the UBX2 truncated-IARS1 and LARS1 UNE-L was analyzed by gel filtration. The data are representative of two independent experiments. **b** A mixture of IARS1, LARS1 and the catalytic domain (residues 176–706) of EARS1 was analyzed by gel filtration. The elution positions of standard size markers are indicated by arrowheads. The data are representative of four independent experiments. **a, b** Source data are provided as a Source Data file. **c** A schematic model of the EARS1 (blue), IARS1 (green), and LARS1 (white) ternary complex.

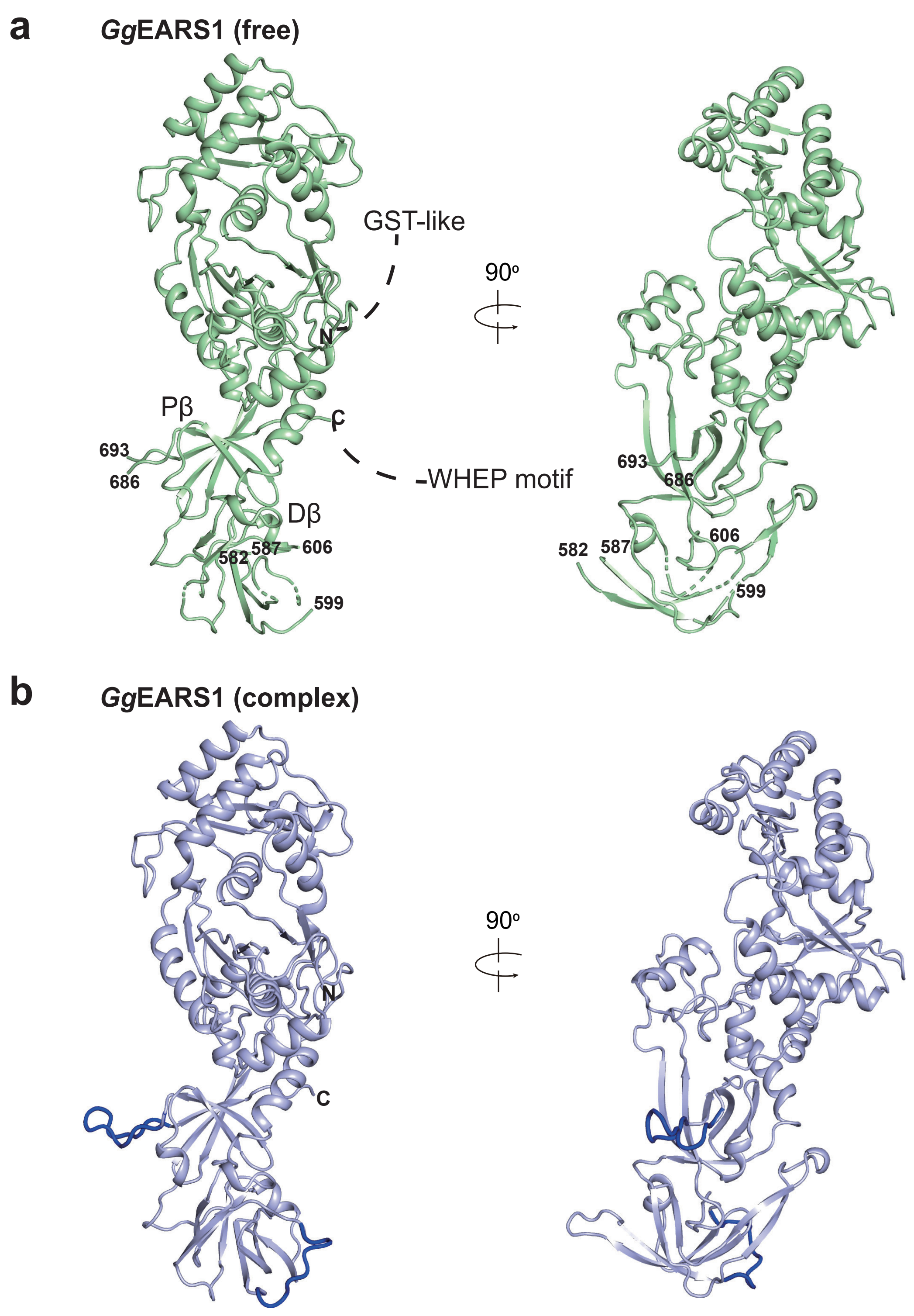

Supplementary Figure 2 Overall structures of apo *Gg*EARS1 and EARS1 in the EARS1–IARS1 complex. a The 2.5 Å crystal structure of apo *Gg*EARS1. The disordered regions including a hairpin loop (residues 687–692) in the Pβ domain and residues 583–586 and 600–605 in the Dβ domain are labeled. The GST-like domain and WHEP motif linked to the N- and C-terminal ends of EARS1, respectively, are truncated to aid crystallization. **b** Structure of the core domain of *Gg*EARS1 in the EARS1–IARS1 complex. The disordered regions in the apo *Gg*EARS1 structure are ordered in the EARS1–IARS1 complex, and shown in blue.

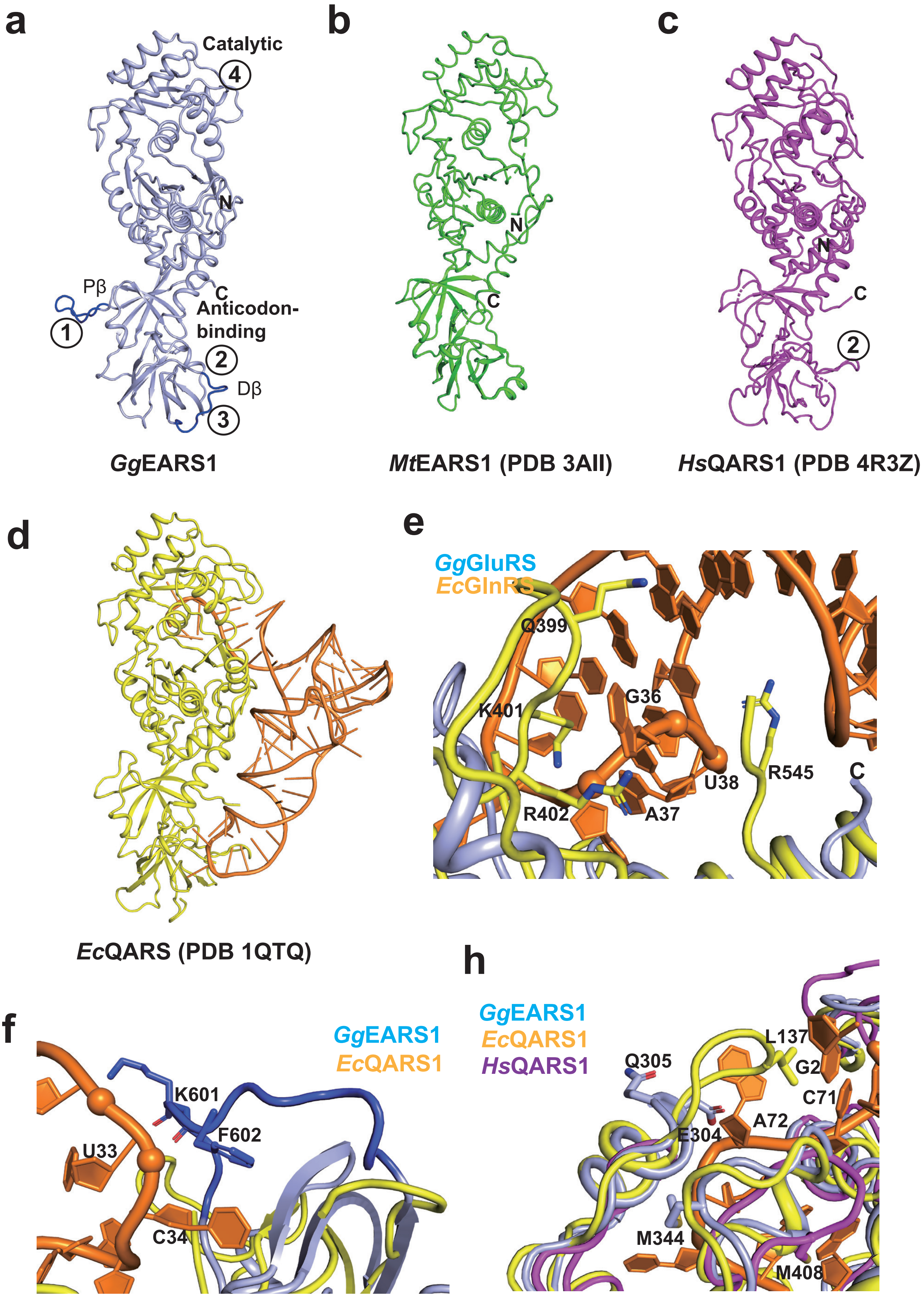

Supplementary Figure 3 Structural differences between *Gg*EARS1, archaeal EARS1 and bacterial and mammalian QARS1s. a A ribbon representation of the *Gg*EARS1 structure.

Structural differences between EARS1 and QARS1 in four loops are indicated by circles. **b**, **c** Structures of EARS1 and QARS1: *M. thermautotrophicus* EARS1 (PDB 3AII[1], middle; **b**) and *H. sapiens* QARS1 (PDB 4R3Z[2], right; **c**). **d** A ribbon representation of the *E. coli* QARS1 (yellow)–tRNA complex (PDB 1QTQ[3]). The $tRNA^{Gln}$ molecule in the complex is colored orange. **e** Close-up view of the Dβ domain from *Gg*EARS1 (light blue) and *Ec*QARS1 (yellow)–tRNA (orange) complex. Residues in *Gg*EARS1 that could contribute to the binding of tRNA are indicated by ball-and-stick. **f** Close-up view of the tRNA-binding surface from *Gg*EARS1 (light blue) and *Ec*QARS1 (yellow)–tRNA (orange) complex. Residues in *Ec*QARS1 that recognize the anticodon bases are indicated by ball-and-stick. **g** Close-up view of the acceptor arm-binding sites from *Gg*EARS1 (light blue), *Hs*QARS1 (magenta) and *Ec*QARS1 (yellow)–tRNA (orange) complex. Residues in β-turn of *Ec*QARS1 and *Gg*EARS1, or residues in loop S5–H7 of *Gg*EARS1 and *Hs*QARS1 are shown.

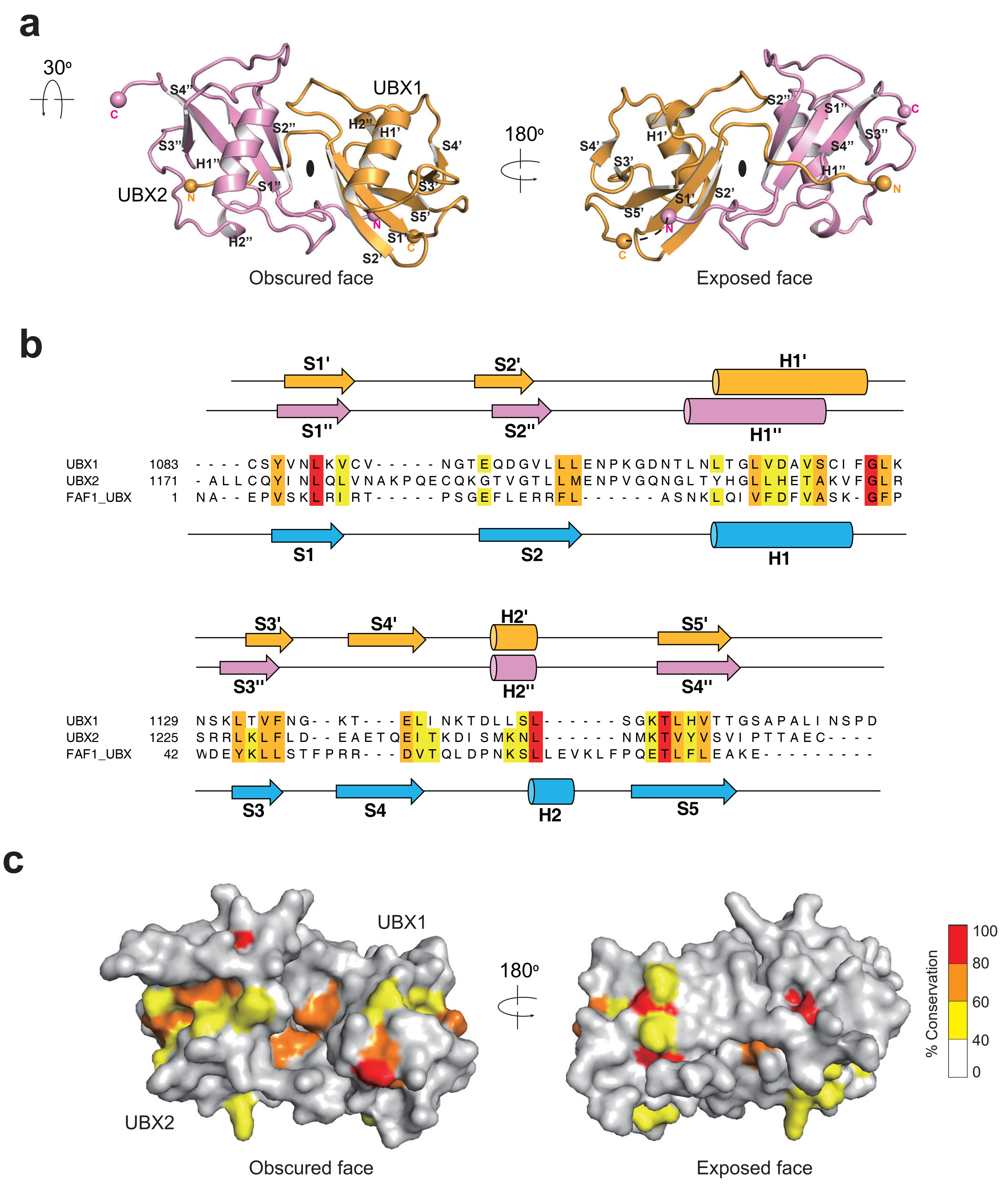

Supplementary Figure 4 IARS1 UNE-I folds into tandem UBX domains. a Structure of the UNE-I UBX domains. Both the obscured face and exposed face are shown. The pseudo 2-

fold symmetry axis between UBX1 (orange) and UBX2 (magenta) is shown by a black circle. The dashed line indicates the disordered linker region between UBX1 and UBX2. **b** Structure-based sequence alignments of UBX1, UBX2, and FAS associated factor 1 (FAF1) UBX domain (IPR033043). Strictly conserved residues are colored red, highly conserved residues (80%) are colored orange, and less conserved residues (60%) are colored yellow. The secondary structure is displayed at the top and bottom of the sequence. **c** A surface representation of the UBX domains with the degree of sequence conservation denoted by the same colors as in **b**.

a

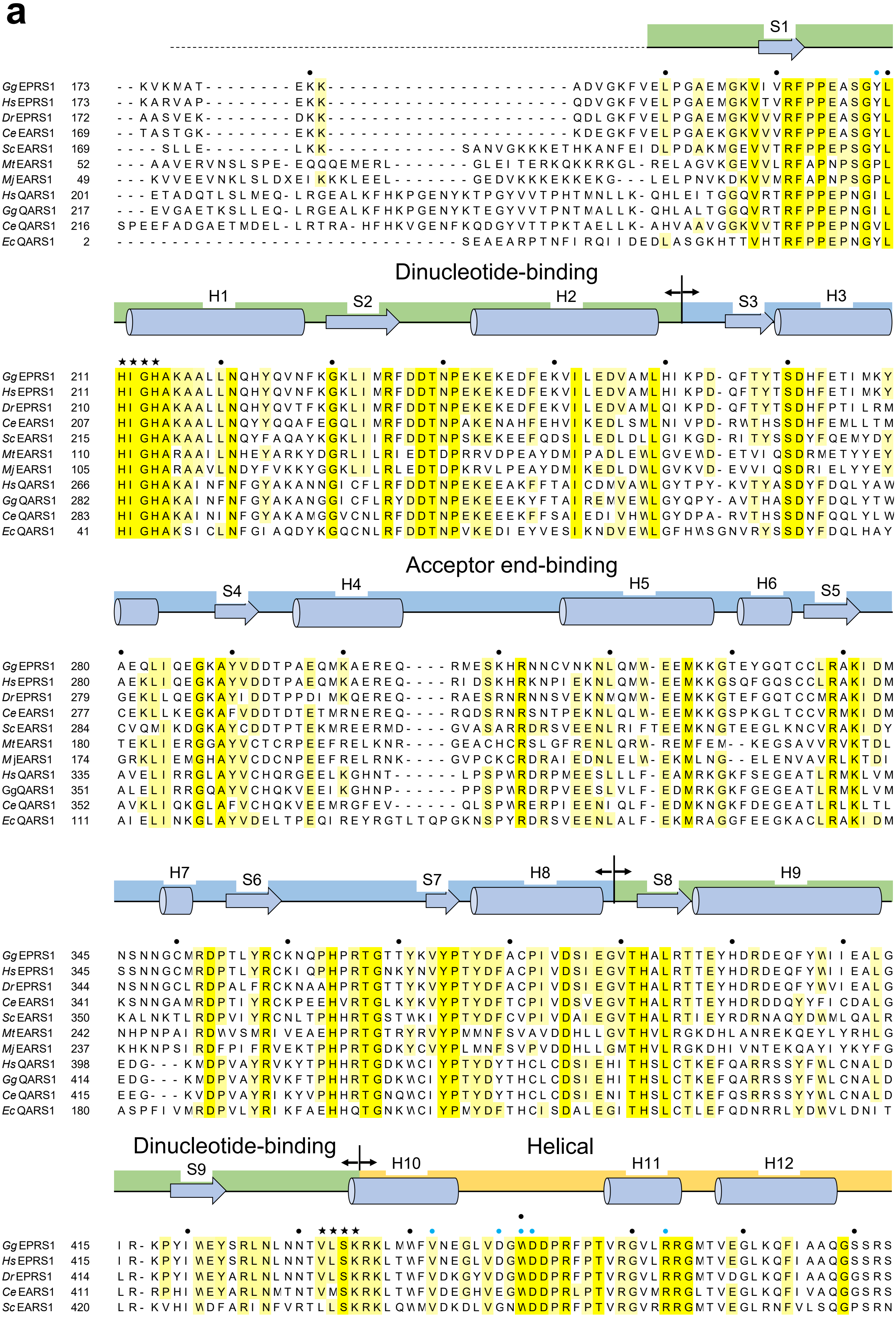

Dinucleotide-binding
Acceptor end-binding
Dinucleotide-binding
Helical
S1
H1
S2
H2
S3
H3
S4
H4
H5
H6
S5
H7
S6
S7
H8
S8
H9
S9
H10
H11
H12

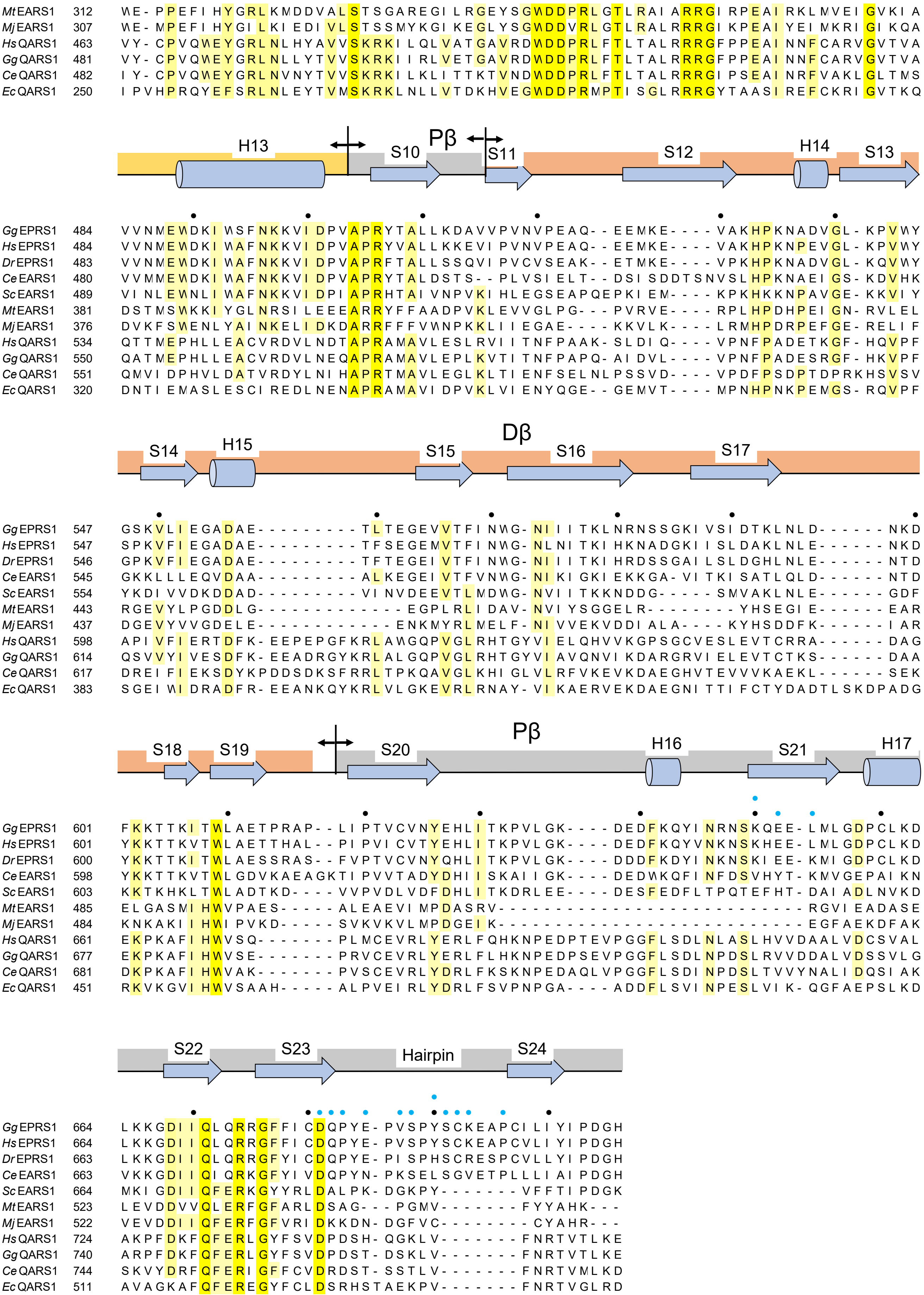
Mt EARS1 312 WE-PPEFIHYGRLKMDDVALSTSGAREGILRGEYSGWDDPRLGTLRAIARRGIRPEAIRKLMVEIGVKIA
Mj EARS1 307 WE-MPEFIHYGILKIEDIVLSTSSMYKGIKEGLYSGWDDVRLGTLRALRRRGIKPEAIYEIMKRIGIKQA
Hs QARS1 463 VY-CPVQWEYGRLNLHYAVVSKRKILQLVATGAVRDWDDPRLFTLTALRRRGFPPEAINNFCARVGVTVA
Gg QARS1 481 VY-CPVQWEYGRLNLLYTVVSKRKIIRLVETGAVRDWDDPRLFTLTALRRRGFPPEAINNFCARVGVTVA
Ce QARS1 482 IY-CPVQWEYGRLNVNYTVVSKRKILKLITTKTVNDWDDPRLFTLTALRRRGIPSEAINRFVAKLGLTMS
Ec QARS1 250 IPVHPRQYEFSRLNLEYTVMSKRKLNLLVTDKHVEGWDDPRMPTISGLRRRGYTAASIREFCKRIGVTKQ
H13
Pβ
S10
S11
S12
H14
S13
Gg EPRS1 484 VVNMEWDKIWSFNKKVIDPVAPRYTALLKDAVVPVNVPEAQ--EEMKE----VAKHPKNADVGL-KPVWY
Hs EPRS1 484 VVNMEWDKIWAFNKKVIDPVAPRYVALLKKEVIPVNVPEAQ--EEMKE----VAKHPKNPEVGL-KPVWY
Dr EPRS1 483 VVNMEWDKIWAFNKKVIDPVAPRFTALLSSQVIPVCVSEAK--ETMKE----VPKHPKNADVGL-KQVWY
Ce EARS1 480 VVMMEWDKIWAFNKKVIDPVAPRYTALDSTS--PLVSIELT--DSISDDTSNVSLHPKNAEIGS-KDVHK
Sc EARS1 489 VINLEWNLIWAFNKKVIDPIAPRHTAIVNPVKIHLEGSEAPQEPKIEM----KPKHKKNPAVGE-KKVIY
Mt EARS1 381 DSTMSWKKIYGLNRSILEEEARRYFFAADPVKLEVVGLPG---PVRVE----RPLHPDHPEIGN-RVLEL
Mj EARS1 376 DVKFSWENLYAINKELIDKDARRFFFVWNPKKLIIEGAE----KKVLK----LRMHPDRPEFGE-RELIF
Hs QARS1 534 QTTMEPHLLEACVRDVLNDTAPRAMAVLESLRVIITNFPAAK-SLDIQ----VPNFPADETKGF-HQVPF
Gg QARS1 550 QATMEPHLLEACVRDVLNEQAPRAMAVLEPLKVTITNFPAPQ-AIDVL----VPNFPADESRGF-HKVPF
Ce QARS1 551 QMVIDPHVLDATVRDYLNIHAPRTMAVLEGLKLTIENFSELNLPSSVD----VPDFPSDPTDPRKHSVSV
Ec QARS1 320 DNTIEMASLESCIREDLNENAPRAMAVIDPVKLVIENYQGE--GEMVT----MPNHPNKPEMGS-RQVPF
Dβ
S14
H15
S15
S16
S17
Gg EPRS1 547 GSKVLIEGADAE----------TLTEGEVVTFINWG-NIIITKLNRNSSGKIVSIDTKLNLD------NKD
Hs EPRS1 547 SPKVFIEGADAE----------TFSEGEMVTFINWG-NLNITKIHKNADGKIISLDAKLNLE------NKD
Dr EPRS1 546 GPKVFIEGADAE----------TFTEGEIVTFINWG-NIIITKIHRDSSGAILSLDGHLNLE------NTD
Ce EARS1 545 GKKLLLEQVDAA----------ALKEGEIVTFVNWG-NIKIGKIEKKGA-VITKISATLQLD------NTD
Sc EARS1 554 YKDIVVDKDDAD----------VINVDEEVTLMDWG-NVIITKKNDDG-----SMVAKLNLE------GDF
Mt EARS1 443 RGEVYLPGDDLG--------------EGPLRLIDAV-NVIYSGGELR--------YHSEGIE------EAR
Mj EARS1 437 DGEVYVVGDELE-------------ENKMYRLMELF-NIVVEKVDDIALA----KYHSDDFK------IAR
Hs QARS1 598 APIVFIERTDFK-EEPEPGFKRLAWGQPVGLRHTGYVIELQHVVKGPSGCVESLEVTCRRA------DAG
Gg QARS1 614 QSVVYIVESDFK-EEADRGYKRLALGQPVGLRHTGYVIAVQNVIKDARGRVIELEVTCTKS------DAA
Ce QARS1 617 DREIFIEKSDYKPDDSDKSFRRLTPKQAVGLKHIGLVLRFVKEVKDAEGHVTEVVVKAEKL------SEK
Ec QARS1 383 SGEIWIDRADFR-EEANKQYKRLVLGKEVRLRNAY-VIKAERVEKDAEGNITTIFCTYDADTLSKDPADG
S18
S19
S20
Pβ
H16
S21
H17
Gg EPRS1 601 FKKTTKITWLAETPRAP--LIPTVCVNYEHLITKPVLGK----DEDFKQYINRNSKQEE-LMLGDPCLKD
Hs EPRS1 601 YKKTTKVTWLAETTHAL--PIPVICVTYEHLITKPVLGK----DEDFKQYVNKNSKHEE-LMLGDPCLKD
Dr EPRS1 600 YKKTTKITWLAESSRAS--FVPTVCVNYQHLITKPVLGK----DDDFKDYINKNSKIEE-KMIGDPCLKD
Ce EARS1 598 YKKTTKVTWLGDVKAEAGKTIPVVTADYDHIISKAIIGK----DEDWKQFINFDSVHYT-KMVGEPAIKN
Sc EARS1 603 KKTKHKLTWLADTKD----VVPVDLVDFDHLITKDRLEE----DESFEDFLTPQTEFHT-DAIADLNVKD
Mt EARS1 485 ELGASMIHWVPAES-----ALEAEVIMPDASRV---------------------------RGVIEADASE
Mj EARS1 484 KNKAKIIHWIPVKD-----SVKVKVLMPDGEIK---------------------------EGFAEKDFAK
Hs QARS1 661 EKPKAFIHWVSQ-------PLMCEVRLYERLFQHKNPEDPTEVPGGFLSDLNLASLHVVDAALVDCSVAL
Gg QARS1 677 EKPKAFIHWVSE-------PRVCEVRLYERLFLHKNPEDPSEVPGGFLSDLNPDSLRVVDDALVDSSVLG
Ce QARS1 681 DKPKAFIHWVAK-------PVSCEVRLYDRLFKSKNPEDAQLVPGGFLSDINPDSLTVVYNALIDQSIAK
Ec QARS1 451 RKVKGVIHWVSAAH-----ALPVEIRLYDRLFSVPNPGA----ADDFLSVINPESLVIK-QGFAEPSLKD
S22
S23
Hairpin
S24
Gg EPRS1 664 LKKGDIIQLQRRGFFICDQPYE-PVSPYSCKEAPCILIYIPDGH
Hs EPRS1 664 LKKGDIIQLQRRGFFICDQPYE-PVSPYSCKEAPCVLIYIPDGH
Dr EPRS1 663 LKKGDIIQLQRRGFYICDQPYE-PISPHSCRESPCVLLYIPDGH
Ce EARS1 663 VKKGDIIQIQRKGFYIVDQPYN-PKSELSGVETPLLLIAIPDGH
Sc EARS1 664 MKIGDIIQFERKGYYRLDALPK-DGKPY--------VFFTIPDGK
Mt EARS1 523 LEVDDVVQLERFGFARLDSAG---PGMV--------FYYAHK---
Mj EARS1 522 VEVDDIIQFERFGFVRIDKKDN-DGFVC---------CYAHR---
Hs QARS1 724 AKPFDKFQFERLGYFSVDPDSH-QGKLV--------FNRTVTLKE
Gg QARS1 740 ARPFDKFQFERLGYFSVDPDST-DSKLV--------FNRTVTLKE
Ce QARS1 744 SKVYDRFQFERIGFFCVDRDST-SSTLV--------FNRTVMLKD
Ec QARS1 511 AVAGKAFQFEREGYFCLDSRHSTAEKPV--------FNRTVGLRD

b

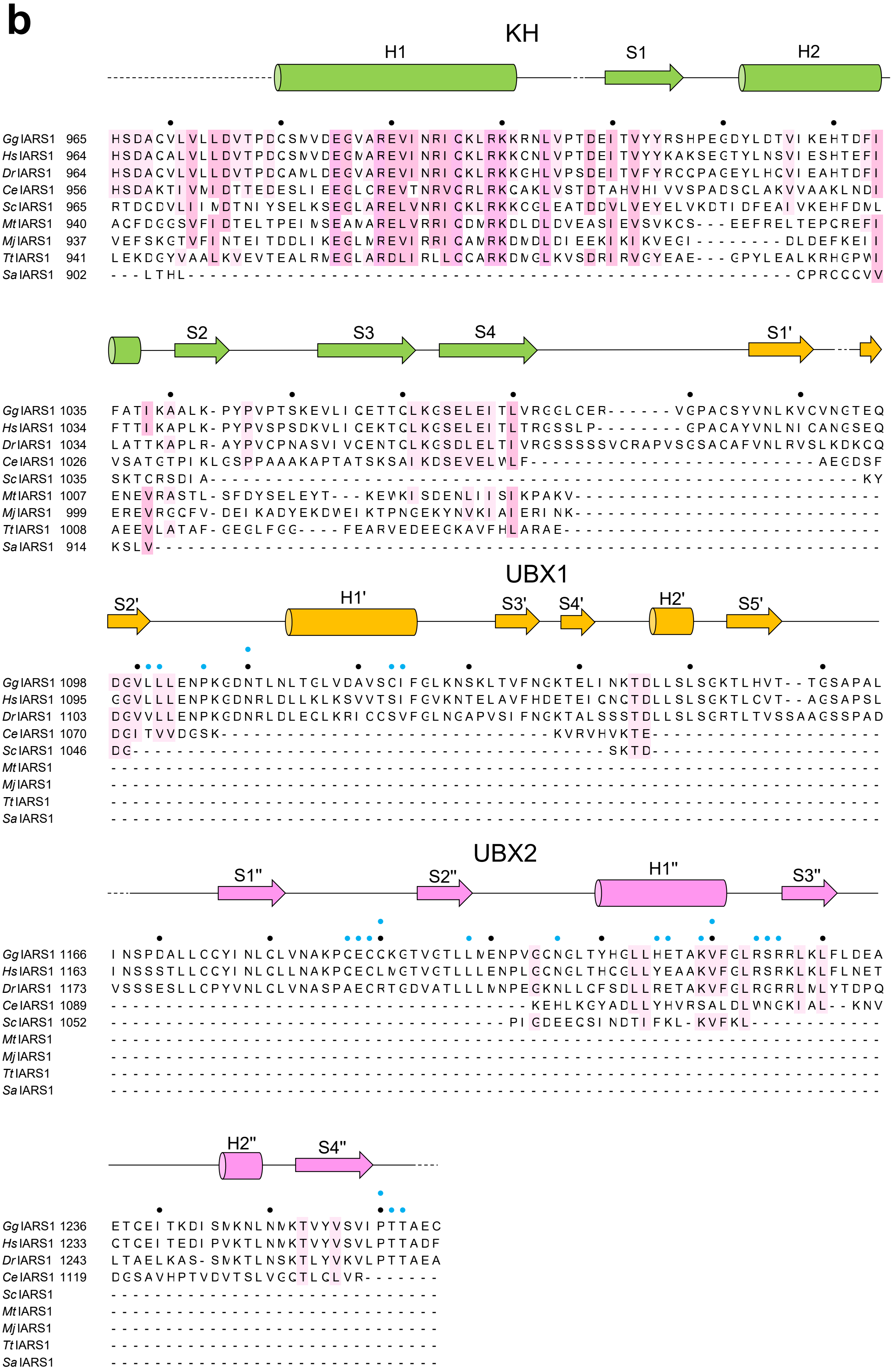
KH
H1
S1
H2
GgIARS1 965 HSDAGVLVLLDVTPDGSMVDEGVAREVINRIGKLRKKRNLVPTDEITVYYRSHPEGDYLDTVIKEHTDFI
HsIARS1 964 HSDAGALVLLDVTPDGSMVDEGMAREVINRIGKLRKKGNLVPTDEITVYYKAKSEGTYLNSVIESHTEFI
DrIARS1 964 HSDAGVLVLLDVTPDGAMLDEGVAREVINRIGKLRKKGHLVPSDEITVFYRGGPAGEYLHGVIEAHTDFI
CeIARS1 956 HSDAKTIVMIDTTEDESLIEEGLGREVTNRVGRLRKGAKLVSTDTAHVHIVVSPADSGLAKVVAAKLNDI
ScIARS1 965 RTDGDVLIIMDTNIYSELKSEGLARELVNRIGKLRKKGGLEATDDVLVEYELVKDTIDFEAIVKEHFDML
MtIARS1 940 AGFDGGSVFIDTELTPEIMSEAMARELVRRIGDMRKDLDLDVEASIEVSVKCS---EEFRELTEPGREFI
MjIARS1 937 VEFSKGTVFINTEITDDLIKEGLMREVIRRIGAMRKDMDLDIEEKIKIKVEGI--------DLDEFKEII
TtIARS1 941 LEKDGYVAALKVEVTEALRMEGLARDLIRLLGGARKDMGLKVSDRIRVGYEAE---GPYLEALKRHGPWI
SaIARS1 902 ---LTHL---CPRCGGVV
S2
S3
S4
S1'
GgIARS1 1035 FATIKAALK-PYPVPTSKEVLIGETTGLKGSELEITLVRGGLCER------VGPACSYVNLKVCVNGTEQ
HsIARS1 1034 FTTIKAPLK-PYPVSPSDKVLIGEKTGLKGSELEITLTRGSSLP--------GPACAYVNLNICANGSEQ
DrIARS1 1034 LATTKAPLR-AYPVCPNASVIVGENTGLKGSDLELTIVRGSSSSSVCRAPVSGSACAFVNLRVSLKDKGQ
CeIARS1 1026 VSATGTPIKLGSPPAAAKAPTATSKSAIKDSEVELWLF--------------------------AEGDSF
ScIARS1 1035 SKTGRSDIA---KY
MtIARS1 1007 ENEVRASTL-SFDYSELEYT---KEWKISDENLIISIKPAKV--------------------------
MjIARS1 999 EREVRGGFV-DEIKADYEKDWEIKTPNGEKYNVKIAIERINK--------------------------
TtIARS1 1008 AEEVLATAF-GEGLFGG----FEARVEDEEGKAVFHLARAE--------------------------
SaIARS1 914 KSLV---
UBX1
S2'
H1'
S3'
S4'
H2'
S5'
GgIARS1 1098 DGVLLLENPKGDNTLNLTGLVDAVSCIFGLKNSKLTVFNGKTELINKTDLLSLSGKTLHVT--TGSAPAL
HsIARS1 1095 GGVLLLENPKGDNRLDLLKLKSVVTSIFGVKNTELAVFHDETEIGNGTDLLSLSGKTLCVT--AGSAPSL
DrIARS1 1103 DGVVLLENPKGDNRLDLEGLKRIGGSVFGLNGAPVSIFNGKTALSSSTDLLSLSGRTLTVSSAAGSSPAD
CeIARS1 1070 DGITVVDGSK----------------------------KVRVHVKTE--------------------
ScIARS1 1046 DG------------------------------------SKTD--------------------
MtIARS1 --
MjIARS1 --
TtIARS1 --
SaIARS1 --
UBX2
S1"
S2"
H1"
S3"
GgIARS1 1166 INSPDALLCGYINLGLVNAKPGECGKGTVGTLLMENPVGGNGLTYHGLLHETAKVFGLRSRRLKLFLDEA
HsIARS1 1163 INSSSTLLCGYINLGLLNAKPGECLMGTVGTLLLENPLGGNGLTHGGLLYEAAKVFGLRSRKLKLFLNET
DrIARS1 1173 VSSSESLLCPYVNLGLVNASPAECRTGDVATLLLMNPEGKNLLGFSDLLRETAKVFGLRGRRLMLYTDPQ
CeIARS1 1089 --KEHLKGYADLLYHVRSALDLWNGKIAL--KNV
ScIARS1 1052 ----------------------------------PIGDEEGSINDTIFKL-KVFKL------------
MtIARS1 --
MjIARS1 --
TtIARS1 --
SaIARS1 --
H2"
S4"
GgIARS1 1236 ETGEITKDISMKNLNMKTVYVSVIPTTAEC
HsIARS1 1233 GTGEITEDIPVKTLNMKTVYVSVLPTTADF
DrIARS1 1243 LTAELKAS-SMKTLNSKTLYVKVLPTTAEA
CeIARS1 1119 DGSAVHPTVDVTSLVGGTLGLVR------
ScIARS1 ------------------------------
MtIARS1 ------------------------------
MjIARS1 ------------------------------
TtIARS1 ------------------------------
SaIARS1 ------------------------------

Supplementary Figure 5 Sequence alignments of EARS1 and QARS1 or IARS1. a Structure-based sequence alignments of EARS1 and QARS1 orthologs. The organisms of EARS1 are *Gallus gallus* (Accession, XP_015139136), *Homo sapiens* (NP_004437), *Danio rerio* (NP_001275581), *Caenorhabditis elegans* (NP_001366832), *Saccharomyces cerevisiae* (NP_011269), *Methanothermobacter thermautotrophicus* (WP_010875691) and *Methanocaldococcus jannaschii* (WP_010870894). The organisms of QARS1 are *H. sapiens* (Accession, NP_001259002), *G. gallus* (NP_001012800), *Caenorhabditis elegans* (NP_001255843) and *Escherichia. coli* (NP_308737). The secondary structure of *Gg*EARS1 is displayed above the sequences. The locations and functions of the key residues are highlighted and annotated. H, α-helix; S, β-sheet; black star, signature motif; blue circle, IARS1 interaction residues. Every tenth residue is marked with a black dot. **b** Structure-based sequence alignments of IARS1 orthologs. The organisms of IARS1 are *G. gallus* (Uniprot ID, A0A3Q2UG33), *H. sapiens* (P41252), *D. rerio* (F1QSY7), *C. elegans* (Q21926), *S. cerevisiae* (P09436), *M. thermautotrophicus* (O27428), *M. jannaschii* (Q58357), *Thermus thermophilus* (P56690), and *Staphylococcus aureus* (P41972). The secondary structure of *Gg*IARS1 is displayed above the sequences. The locations and functions of the key residues are highlighted and annotated.

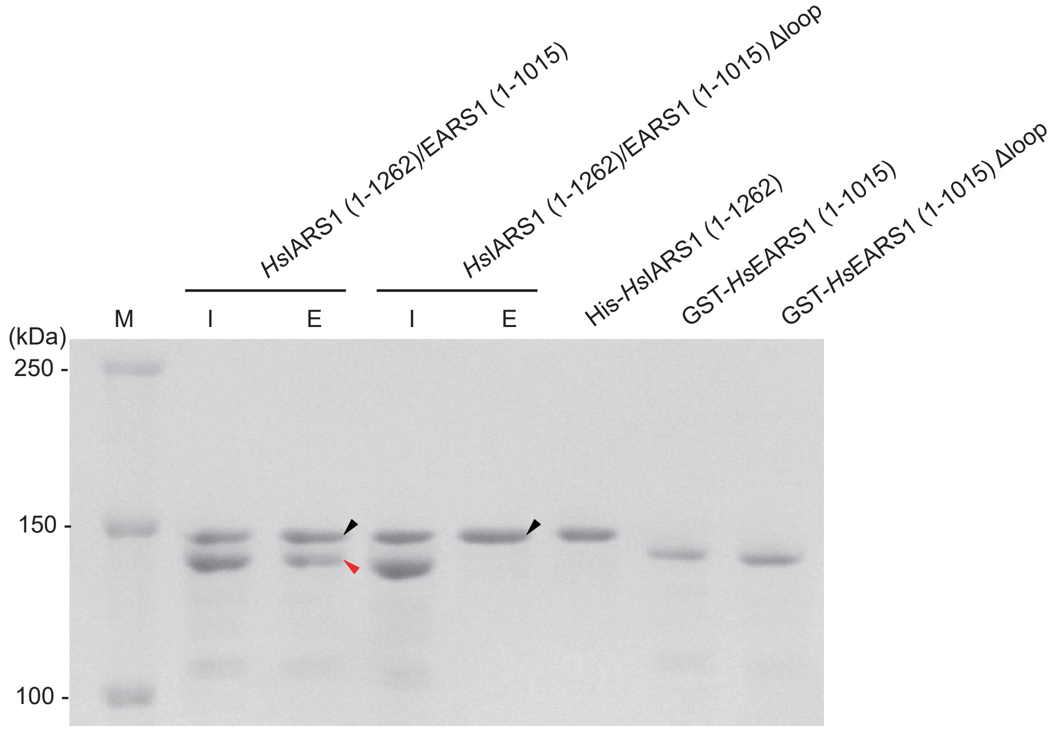

Supplementary Figure 6 The hairpin loop of EARS1 is the primary binding site for IARS1. Affinity pull-down analysis of the interaction between IARS1 and the hairpin-deleted EARS1 with three WHEP repeats. The input (I) and eluate (E) fractions were analyzed by SDS-PAGE and Coomassie Blue staining. Black and red arrowheads indicate eluted His-IARS1 and GST-EARS1, respectively. The data are representative of three independent experiments. Source data are provided as a Source Data file.

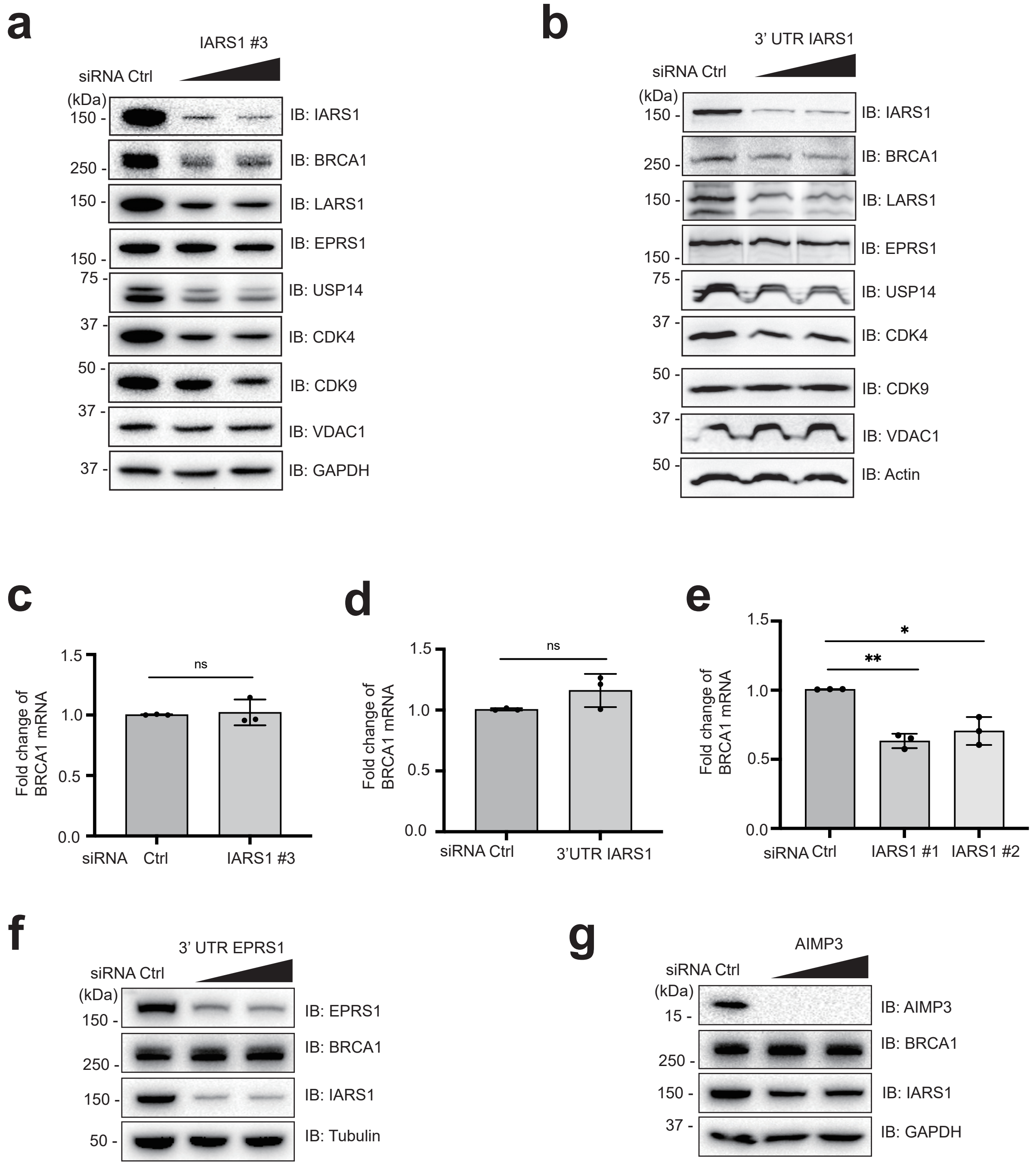

Supplementary Figure 7 Effects of IARS1 depletion on the stabilities of putative binding proteins. **a**, **b** Protein extracts from HeLa cells transfected with control siRNA or siRNA^{IARS1}#3 (**a**) and 3’UTR-specific siRNA^{IARS1} (**b**) were immunoblotted with anti-IARS1, anti-BRCA1, anti-LARS1, anti-EPRS, anti-USP14, anti-CDK4, anti-CDK9, anti-VDAC1, and anti-GAPDH or actin (loading control) antibodies. Transfections were performed with two siRNAs^{IARS1} at 40nM or 80nM. The data are representative of three independent experiments.

Supplementary Figure 8 Nuclear localization of IARS1. a The subcellular localization of overexpressed Flag-tagged IARS1 in HeLa cells was examined by immunofluorescence microscopy. Nuclei were stained with DAPI (blue). Scale bars represent 10 μm. **b** HeLa cells were transfected with Flag-tagged IARS1 for cytosolic and nuclear fractionation analysis. Lamin A/C and GAPDH were used as nuclear and cytoplasmic markers, respectively. WCL, whole-cell lysates. The data are representative of three independent experiments. Source data are provided as a Source Data file.

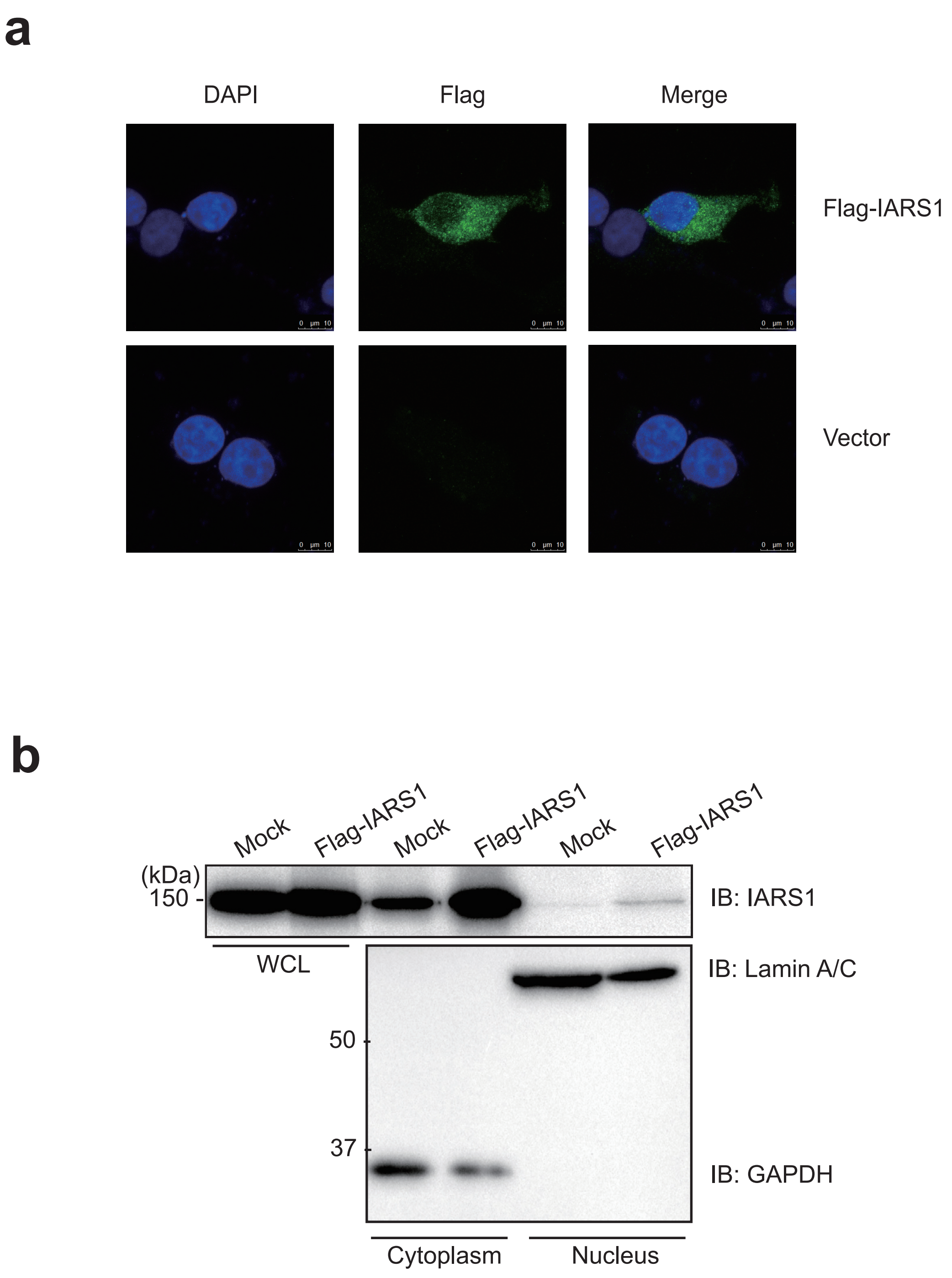

Supplementary Figure 8 Nuclear localization of IARS1. a The subcellular localization of overexpressed Flag-tagged IARS1 in HeLa cells was examined by immunofluorescence microscopy. Nuclei were stained with DAPI (blue). Scale bars represent 10 μm. **b** HeLa cells were transfected with Flag-tagged IARS1 for cytosolic and nuclear fractionation analysis. Lamin A/C and GAPDH were used as nuclear and cytoplasmic markers, respectively. WCL, whole-cell lysates. The data are representative of three independent experiments. Source data are provided as a Source Data file.

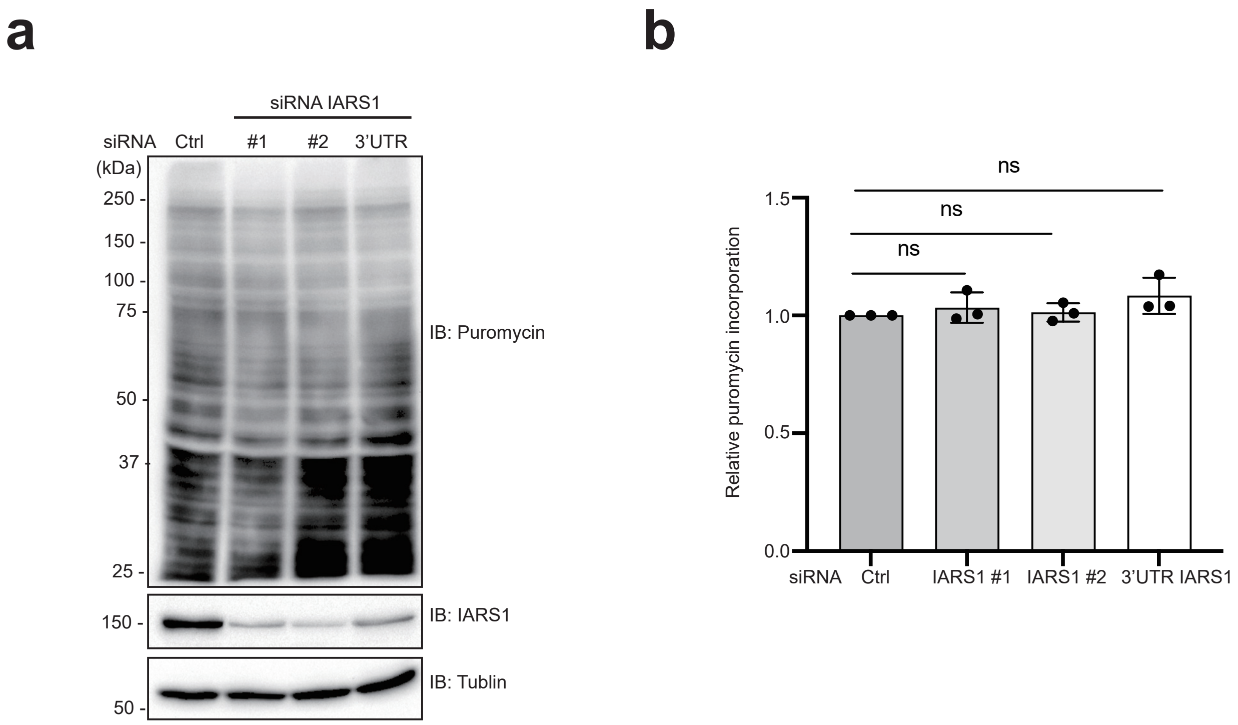

Supplementary Figure 9 Effects of IARS1 depletion on *de novo* protein synthesis. a HeLa cells were transfected with control siRNA or three $siRNA^{IARS1}$ and treated with 1 μM puromycin for 30 min to label nascent peptides. Nascent peptides labeled with puromycin were detected by immunoblotting with an anti-puromycin antibody. Tubulin served as a loading control. **b** Quantification of puromycin labeled proteins in (**a**). Graph shows relative amounts of puromycin incorporation. Error bars represent the mean values ± SD (n=3). ns, not significant by the analysis of two-tailed paired Student's t-test. Source data are provided as a Source Data file.

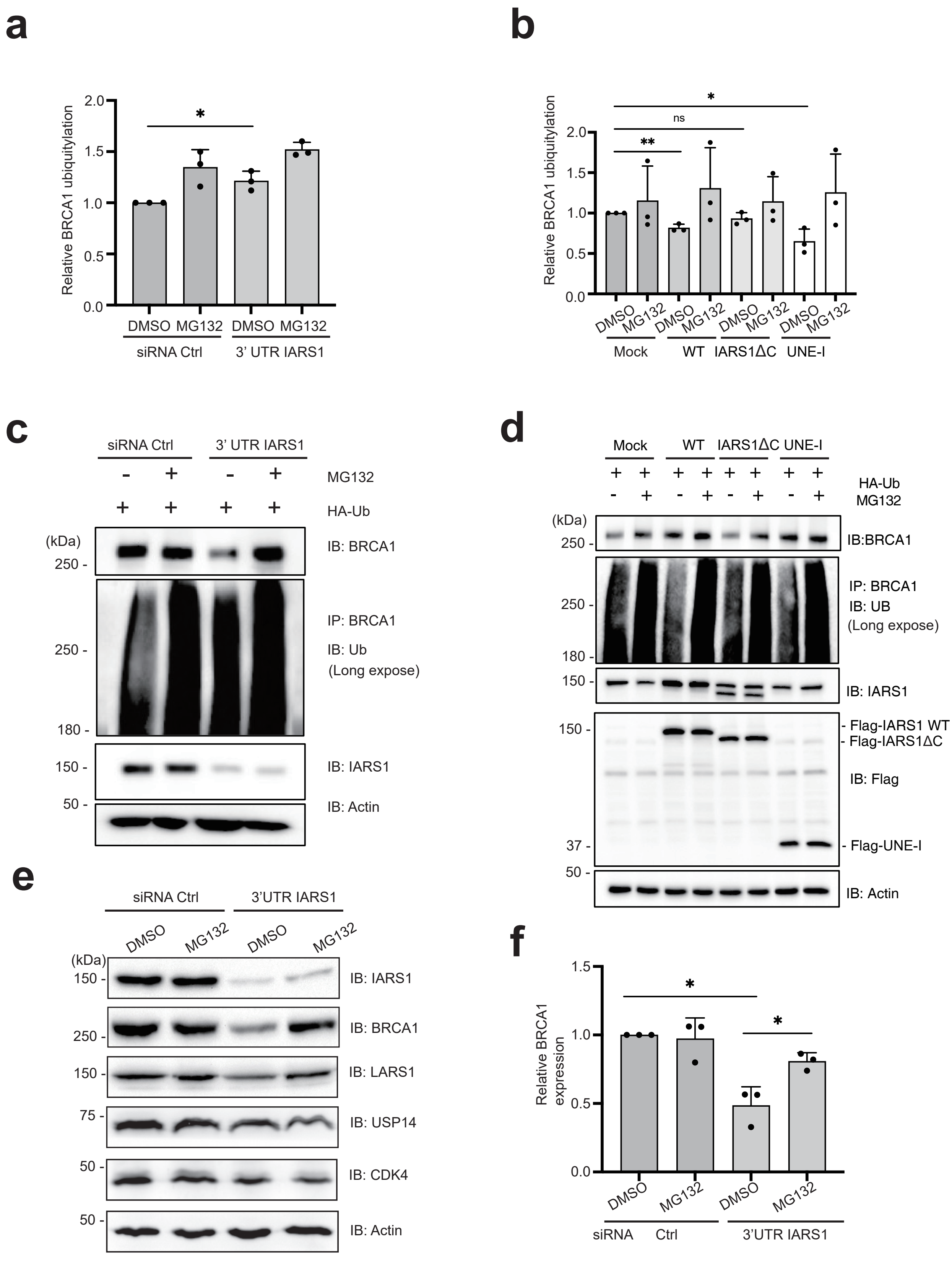

Supplementary Figure 10 The UBX domains of IARS1 are responsible for BRCA1 stability. a Quantification of ubiquitylated BRCA1 (Fig. 4d) in HEK293T cells transfected with control siRNA or 3'UTR-specific siRNA^{IARS1}. The mean values ± SD of three independent (siRNA Ctrl and 3'UTR-specific siRNA^{IARS1}) experiments are shown.

b Quantification of ubiquitylated BRCA1 (Fig. 4e) in HEK293T cells transfected with full-length IARS1, IARS1ΔC, and IARS1 UNE-I alone. The mean values ± SD of three independent (WT, IARS1ΔC, and IARS1 UNE-I) experiments are shown. $^{*}P < 0.05$, $^{**}P < 0.01$, ns, not significant by the analysis of two-tailed paired Student's t-test in **a** and **b**. **c** *In vivo* ubiquitylation assay of BRCA1 in HEK293T cells transfected with control siRNA or 3'UTR-specific $siRNA^{IARS1}$. **d** *In vivo* ubiquitylation assay of BRCA1 in HEK293T cells transfected with full-length IARS1, IARS1ΔC, and IARS1 UNE-I alone. **e** HeLa cells transfected with control siRNA or 3'UTR-specific $siRNA^{IARS1}$ were treated with 10 μM MG132 for 4 hr and analyzed by immunoblotting with the indicated antibodies. **f** Quantification of BRCA1 proteins in **e**. The mean values ± SD of three independent (siRNA Ctrl and 3'UTR-specific $siRNA^{IARS1}$) experiments are shown. $^{*}P < 0.05$, $^{**}P < 0.01$ by the analysis of two-tailed paired Student's t-test. Source data are provided as a Source Data file.

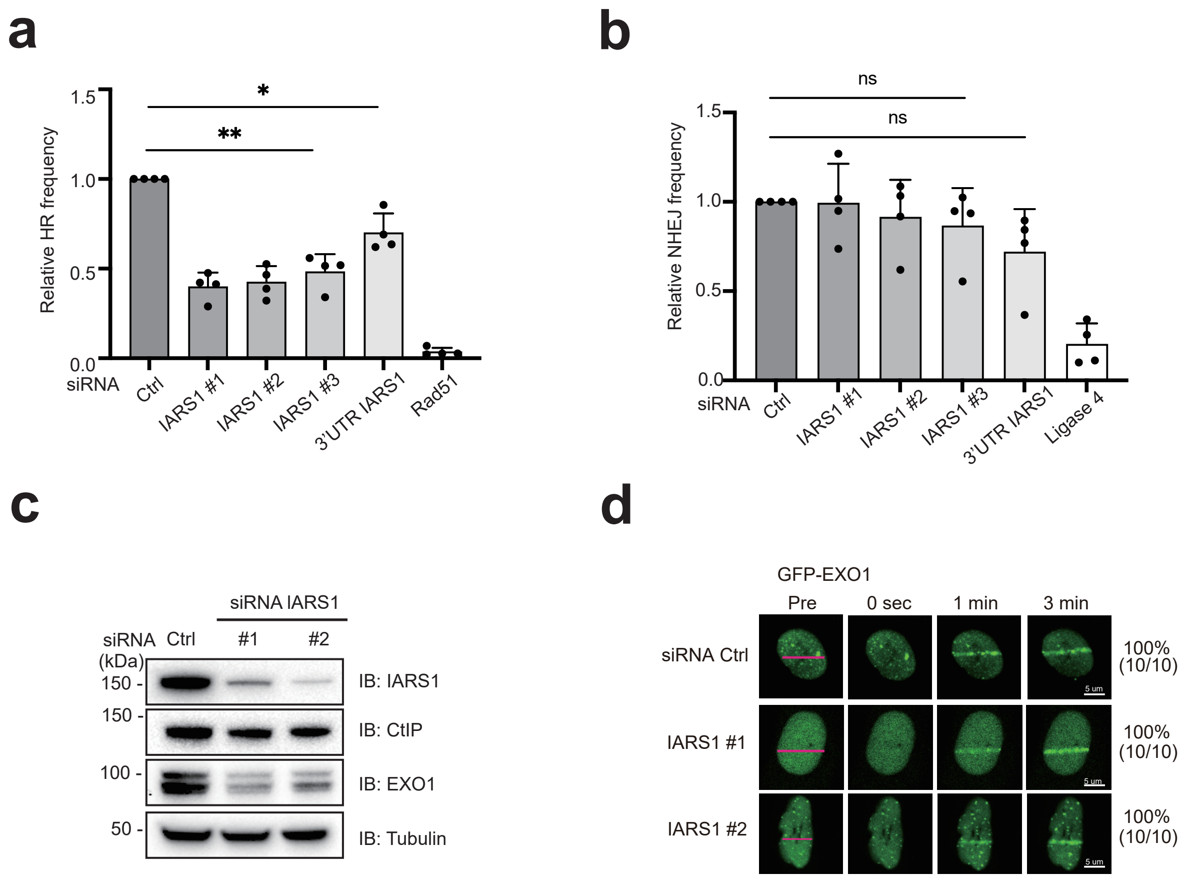

Supplementary Figure 11 Effects of IARS1 depletion on the stabilities of DNA damage response proteins. a I-*Sce*I-DSB-induced HR efficiencies were determined in DR-U2OS cells upon treatment with control siRNA, three siRNA^{IARS1}, or siRNA^{Rad51}. **b** Efficiency of NHEJ in EJ5-U2OS cells was measured after transfection of control, three siRNA^{IARS1}, or siRNA^{Ligase4}. **a, b** Data are represented as mean values ± SD (n=4, independent cell culture). $*P < 0.05$, $**P < 0.01$, ns, not significant by the analysis of two-tailed paired Student's t-test. **c** Protein extracts from HeLa cells transfected with control siRNA or two siRNAs^{IARS1} were immunoblotted using anti-IARS1, anti-CtIP, anti-EXO1, and anti-tubulin (loading control) antibodies. **d** U2OS cells overexpressing GFP-EXO1 were transfected with control siRNA or two siRNAs^{IARS1} and exposed to laser microirradiation. Laser stripes were examined at the indicated time point. EXO1 recruitment to DSB site was calculated from GFP positive cells over total counted cells. **c, d** The data are representative of three independent experiments. Source data are provided as a Source Data file.

Supplementary Note

Overall structure of EARS1

*Gg*EARS1 forms an elongated shape with the N-terminal region formed by the acceptor end- and dinucleotide-binding domains and the C-terminal anticodon-binding domain formed by the Pβ and Dβ domains. The dinucleotide-binding and Pβ domains are connected by the helical subdomain (residues 433–503), which is formed by four helices in the middle and contacts the D-arm of tRNA. While the electron density map of the N-terminal catalytic domain is clear, both β-barrel subdomains in the C-terminal anticodon-binding domain show significant disorder in the apo *Gg*EARS1 structure, indicating that the regions are highly mobile. The most noticeable disordered regions include a hairpin loop (residues 687–692; loop S23-S24) in the Pβ domain, and residues 583–586 and 600–605 in the Dβ domain. Additional disordered regions include residues 545–546 and 560–563. However, most of the disordered regions become ordered upon complex formation with IARS1.

Structural comparison with archaeal EARS1 and bacterial and mammalian QARS1s

The overall structure of *Gg*EARS1 is highly similar to those of QARS1 and archaeal EARS1, and can be superimposed on those of human QARS1 (PDB 4R3Z)[2], *E. coli* QARS1 (PDB 1QTQ)[3], and archaeal EARS1 (PDB 3AII)[1] with the rms deviation values of 1.8–2.5 Å (386–430 Cα atoms). In the superimposed structures, eukaryotic EARS1 more resembles bacterial and eukaryotic QARS1s than archaeal EARS1 (Supplementary Fig. 3). In all five domains of QARS1 and EARS1, the Dβ domain exhibits the largest deviations, and the selectivity for anticodon binding (Glu or Gln) could be determined by these differences (Supplementary Fig. 3). Three regions of *Gg*EARS1, namely, the IARS1-binding site and anticodon- and acceptor end-binding sites, exhibit most notable differences with respect to QARS1s and archaeal EARS1.

Four loops provide specificity for the binding of $tRNA^{Glu}$ to vertebrate EARS1

The most significant difference between *Gg*EARS1 and archaeal EARS1 or QARS1 is observed in the opposite face of the tRNA-binding interface (Fig. 1 and Supplementary Fig. 3). The β-hairpin loop (residues 684–695) in the Pβ domain is present only in vertebrate EARS1, not in archaeal EARS1 or bacterial or mammalian QARS1. In the apo *Gg*EARS1 structure, this loop is disordered, but in the IARS1-bound state, several main chains of the hairpin loop pair with each other to form a well-ordered β-hairpin structure.

Notable differences are also observed at the U33 and C34 site: the S17-S18 loop in the Dβ domain is present only in *Gg*EARS1 (light blue), not in archaeal EARS1 or bacterial QARS1. The *Hs*QARS1 is more similar to *Gg*EARS1 in this region. Nevertheless, the corresponding loop of human QARS1 is 6 to 10 Å away from the S17-S18 loop of *Gg*EARS1. This loop (Phe601 and Lys602) is proximal to the phosphodiester oxygens of U33 and C34 in the modeled tRNA in the anticodon-binding site (Supplementary Fig. 3e). In *Ec*QARS1 (yellow), no interaction is observed between the amino acid residues and phosphodiester oxygens of U33 and C34. This large difference in the anticodon-binding domain may contribute to the selectivity for binding of $tRNA^{Glu}$ over $tRNA^{Gln}$ by vertebrate EARS1.

There is another notable difference in the tRNA-binding surface, both in the anticodon- and acceptor end-binding sites, which confer the selectivity for $tRNA^{Glu/Gln}$. The anticodon for Glu is CUC, whereas the anticodon for Gln is CUG[1]. In *Ec*QARS1, G36 and A37 in the anticodon loop are sandwiched between the Pβ and Dβ domains (Supplementary Fig. 3f). In the aligned structures with tRNA-bound *Ec*QARS1, the base of G36 is packed by a 10 residue loop containing Gln399, Lys401, and Arg402 of *Ec*QARS1. The loop is also conserved in *Hs*QARS1. Arg402 (or Arg629 in *Hs*QARS1) forms an H-bond with G36. In both *Ec*QARS1 and *Hs*QARS1, the phophodiester oxygens of A37 and U38 are stabilized by Arg545 in the C-terminal loop on the opposite side. Archaeal EARS1 forms a shorter loop in this region, but nevertheless has a weak interaction. Thus, these three proteins could efficiently recognize the purine base at this position. By contrast, *Gg*EARS1 does not have an equivalent loop and no interaction can be formed to stabilize the G36 base (Supplementary Fig. 3f).

In the acceptor arm-binding site, *Ec*QARS1 contains a four residue β-turn (residues 133 to 136), from which Leu136 wedges the G2-C71 base pairs. This β-turn wedge is not present in *Gg*EARS1. Hence, T1-A72 and G2-C71 (the equivalent acceptor arm base pairs) stack against Glu304 and Gln305 of *Gg*EARS1 (Supplementary Fig. 3g). The corresponding region in *Hs*QARS1 shows clear differences. Loop H4-H5 in *Gg*EARS1 is missing in *Hs*QARS1 (magenta). In addition, loop S5-H7 exhibits significant conformational differences and Met344 differs from Met408 in *Hs*EARS1 by 7.8 Å. Archaeal EARS1 also lacks this β-turn. Most of other regions in the acceptor loop are conserved. Collectively, the anticodon-

and acceptor arm-binding sites are clearly distinct in eukaryotic EARS1 and QARS1, demonstrating the specificity of each aaRS.

Supplementary Methods

Crystallization of EARS1 and data collection

The native *Gg*EARS1 crystals were grown at 20°C using the hanging drop vapor diffusion method. The *Gg*EARS1 crystallization buffer contained 85 mM HEPES (pH 7.5), 8.5% isopropanol, 15% glycerol, 40 mM NaF, and 15% PEG 8000. The *Gg*EARS1 mercury (Hg) derivative crystals were obtained by soaking native crystals in 0.5 mM Thimerosal solution for 7 hr and then back-soaking them in crystallization buffer. The resulting crystals belonged to the $P2_1$ space group (a = 89.0 Å, b = 41.8 Å, c = 92.2 Å, β = 100.0º). Diffraction data were collected at –170°C using crystals flash-frozen in crystallization buffer containing 25% glycerol. Diffraction data were collected at 1.0094 Å on Beamline 11C of the Pohang Advanced Light Source (PAL). Datasets of *Gg*EARS1 Hg derivative crystals were processed using the HKL2000 package[5].

Determination of the EARS1 structure

The *Gg*EARS1 structure was determined by a combination of the molecular replacement and single-wavelength anomalous dispersion methods using crystals containing Hg-derivatized *Gg*EARS1. Initially, molecules of the N-terminal UNE-Q domain-deleted catalytic domain of human QARS1 (PDB 4R3Z)[2] were located with the PHASER[6] program, followed by a search for the anticodon-binding domain of human QARS1. Four Hg sites in the asymmetric unit were identified at 3.0 Å resolution using the PHENIX AutoSol[7] program. After flattening the solvent, a high-quality electron density map with a resolution of 2.5 Å was obtained using the PHENIX[7] program. Successive rounds of model building with COOT[8] and refinement with PHENIX[7] were employed to build the complete model. The final refined *Gg*EARS1 model at 2.5 Å resolution (R_{work}/R_{free} of 23.2%/27.4%) had 92.6% of its residues in the most favored region and 2% of residues in the disallowed region (Table 1). The model did not include residues 526, 545, 546, 550, 560–563, 566, 572, 583–586, 600–605, and 687–692 of the anticodon-binding domain.

Data collection using XFELs

The fixed-target serial femtosecond crystallography (FT-SFX) experiment using a nylon mesh and X-ray pulses was conducted at the Nano Crystallography and Coherence Imaging experimental hutch of the Pohang Advanced Light Source X-ray free electron laser [9, 10] (PAL-XFEL). The X-ray energy was 9.7 keV (1.2782 Å) and the photon flux was $\sim 5 \times 10^{11}$

photons per pulse (<20 fs duration). The X-ray pulse was focused to 5 (horizontal) × 8 (vertical) μm^2 (FWHM) using a Kirkpatrick–Baez mirror[11]. The data were collected from a sample chamber filled with helium gas at ambient pressure and room temperature (RT) and were recorded using a MX225-HS detector (Rayonix) with a 4 × 4 binning mode (pixel size: 156 × 156 μm^2). The motion stage for FT-SFX was designed to allow raster scanning of beams up to 60 Hz provided by PAL-XFEL and was custom-built by SmartAct. Piezo SLLV42 and SLL12 (SmartAct) actuators were used for translation in the horizontal and vertical directions, respectively. During raster scanning, the acrylic support containing the nylon mesh sample holder was translated 18 mm in both the vertical and horizontal directions in the sample chamber using a piezo linear stage to avoid illumination of the Al frame by the XFEL pulses. This scanning stage was motioned by a remote control system, without synchronization for the arrival of X-ray pulses. The diffraction images were collected with the raster scanning method from the top to the bottom of the chip, at 50 μm intervals left to right, and then moved 50 μm toward the bottom. The next scan was performed from right to left at 50 m intervals and then moved 50 μm toward the bottom. These raster scanning motions were performed repeatedly until a full dataset had been collected. The velocity of the sample holder mounted in the motion stage was 1.5 mm/s in both the horizontal and vertical directions.

SFX data processing

The diffraction pattern was monitored using OnDA[12], and the hit images, defined as those images containing a minimum of 15 peaks, were filtered using the Cheetah program[13]. The 'peak-finder 8' algorithm was used with key peak-finding parameters, and yielded an average hit-rate of 69.1% (301,437 hits) for *Gg*EARS1–IARS1 crystals. The diffraction images were indexed, integrated, merged, and post-refined using CrystFEL[14]. Indexing was performed using a combination of MOSFLM[15], which uses prior unit cell information for indexing, and DirAx. The detector geometry was refined with several iterations of the detector geometry optimization using CrystFEL's geoptimiser for increasing the indexing rate, and the peaks used for indexing were those outputted from Cheetah[13]. Preliminary indexing results, which were obtained in synchrotron, strongly suggested a primitive orthorhombic lattice, and a subsequent round of indexing was performed. The final indexing rate was 16.9% (50,804 indexed patterns) for *Gg* EARS1–IARS1. The intensities were merged using scaling, partiality, and post-refinement with a partialator in CrystFEL[14].

Cell culture

HEK293T, HeLa, and HCT116 cells were cultured in Dulbecco's Modified Eagle's Medium (Lonza or Hyclone) containing 10% fetal bovine serum (Wellgene or Hyclone) and 100 U/mL penicillin-streptomycin (Gibco). All cell lines were cultured in a humidified incubator with 5% CO_2 at 37°C.

cDNAs

Wild-type EPRS was cloned into the pFlag CMV-2 vector with an N-terminal Flag tag. Flag-tagged EPRS with Ser688-to-Glu (S688E), Ser691-to-Glu (S691E), and Ser688- and Ser691-to-Glu (S688E and S691E) mutations and the hairpin loop-deleted EPRS mutant were generated by extension PCR and inserted into the pFlag CMV-2 vector. Wild-type IARS1, C-terminal truncated IARS1 (residues 1–1081), and IARS1 UNE-I (residues 942–1262) were cloned into the pFlag CMV-2 or pFlag CMV-10 vector with an N-terminal Flag tag. The 3xMyc-BRCA1 cDNA construct was kindly provided by Dr. Junjie Chen.

siRNAs

For RNA interference, the following siRNAs against the coding sequence or 3'UTR of IARS1 were synthesized by Bioneer: siRNA #1, 5'-CUGAAGAGUGUUGUCACUA-3' (sense) and 5'-UAGUGACAACACUCUUCAG-3' (anti-sense); siRNA #2, 5'-CUGUGUAUGUUUCUGUGUU-3' (sense) and 5'-AACACAGAAACAUACACAG-3' (anti-sense); siRNA #3, 5'-GGAAGCCAGAUUGUCAGCCCUCUAU-3' (sense) and 5'-AUAGAGGGCUGACAAUCUGGCUUCC-3' (anti-sense); and 3'UTR siRNA, 5'-CUGAAGAGUGUUGUCACUA-3' (sense) and 5'-UAGUGACAACACUCUUCAG-3' (anti-sense). The following siRNAs against the coding sequence or 3'UTR of AIMP3 or EPRS were synthesized by Bioneer: siRNA AIMP3, 5'-CCAAGUCUAACAGGAUUGACUACUATT-3' (sense) and 5'-UAGUAGUCAAUCCUGUUAGACUUGGTT-3' (anti-sense); and 3'UTR siRNA EPRS, 5'-GCCUAAGUUAACAGUGGAU-3' (sense) and 5'-AUCCACUGUUAACUUAGGC-3' (anti-sense). A non-targeting scrambled siRNA (AccuTarget Negative Control siRNA) was purchased from Bioneer.

Antibodies

An anti-Flag antibody (F1804, 1:2000) was purchased from Sigma-Aldrich. Anti-PARP1 (sc-8007, 1:1,000), anti-GAPDH (sc-47724, 1:5,000), anti-Ub (sc-8017, 1:200), anti-BRCA1 (C20, sc-642, 1:250), anti-IARS1 (sc-271826, 1:1,000), and anti-Lamin A/C (sc-376248, 1:1,000) antibodies were purchased from Santa Cruz Biotechnology. An anti-actin antibody (691001, 1:10,000) was purchased from MP Biomedicals. Anti-USP14 (ab192618, 1:2,000), anti-EPRS (ab31531, 1:2,000), anti-tubulin (ab15568, 1:2,000), and anti-EXO1 (ab95012, 1:1,000) antibodies were purchased from Abcam. An anti-Myc antibody (05-724, 1:1,000) was purchased from Millipore. Anti-CDK9 (2316, 1:1,000) and anti-VDAC (4866, 1:1,000) antibodies were purchased from Cell Signaling. An anti-CDK4 antibody (A304-225A, 1:2,000) was purchased from Bethyl Laboratories.

Transfection of cell lines

Cells were transiently transfected with endotoxin-free plasmid DNAs using PEI (Polysciences) or Lipofectamine 3000 (Invitrogen), and all siRNAs were transfected using Lipofectamine RNAiMAX (Invitrogen), following the manufacturer's instructions. Cells were harvested 48 or 72 hr after transfection for further analysis. To deplete only endogenous IARS1, siRNA targeting the 3'UTR of IARS1 was transfected 5 hr after transfection of a plasmid expressing wild-type or mutant IARS1 protein.

Immunoblot analysis

Cells were washed twice with ice-cold phosphate-buffered saline (PBS) and lysed with RIPA lysis buffer (25 mM Tris pH 7.5, 150 mM NaCl, 1% NP-40, 0.5% sodium deoxycholate, 0.1% SDS, 1 mM PMSF, and an EDTA-free protease inhibitor cocktail). The soluble fraction of the cell lysate was isolated by centrifugation at 13,000 rpm for 20 min at 4°C. Cell lysates were denatured in Laemmli sample buffer and boiled for 5 min at 95°C. Samples were resolved on Tris-glycine SDS-PAGE gels prepared using 40% acrylamide:bis-acrylamide (37:5:1) solution (Bio-Rad). After transfer to activated polyvinyl difluoride or nitrocellulose membranes, the membranes were blocked with 5% skim milk prepared in Tris-buffered saline containing Tween 20 and probed with a target-specific primary antibody. Thereafter, the membranes were incubated with a horseradish peroxidase- or IRdye 800-conjugated secondary antibody. Signals were detected using SuperSignal West Pico Sensitivity Substrate (Thermo Scientific) or a LI-COR Odyssey infrared imaging system.

RNA extraction and cDNA synthesis

siRNA-transfected cells were lysed with 500 µL TRIzol, and total RNA was extracted according to the manufacturer's instructions (Invitrogen). In brief, 100 µL chloroform was added to lysed Hela cells, samples were centrifuged at 17,000 g for 15 min at 4°C, 150 µL aqueous phase was collected, and RNA was precipitated from the aqueous phase by mixing with an equal volume of isopropanol. After incubation for 10 min, samples were centrifuged at 17,000 g for 15 min at 4°C. The RNA pellet was washed twice with 75% (v/v) ethanol, briefly air-dried for 5 min, and dissolved in diethylpyrocarbonate-treated water. The purity and yield of RNA were determined using a NanoDrop 2000 spectrophotometer (Thermo Scientific). For cDNA synthesis, 1 µg total RNA was reverse-transcribed using oligo-dT and the ImProm-II Reverse Transcription System (Promega) according to the manufacturer's instructions. The RT-PCR conditions were one cycle of 25°C for 5 min, 42°C for 60 min, and 72°C for 7 min. For RT-qPCR analysis, each cDNA was diluted 2-fold with nuclease-free water.

Quantitative real-time PCR

The mRNA level of BRCA1 was measured by quantitative real-time PCR using a StepOnePlus Real-Time PCR system (Applied Biosystems) with SYBR Premix Ex Taq (Takara) according to the manufacturer's instructions. The sequences of qPCR primers are shown in Supplementary Table 2. Data were normalized against β-actin expression, and relative expression was calculated using the ΔΔCT method[16].

Immunofluorescence microscopy

To detect the subcellular localization of IARS1, transfected HEK293T cells were seeded on LabTek™ 4-well chamber slides (Thermo Fisher Scientific) at a density of 1×10^4 cells per well. The following day, cells were fixed with 4% paraformaldehyde diluted in PBS for 20 min at RT and washed four times with washing buffer (PBS containing 0.1% Triton X-100). Cells were blocked in blocking buffer (PBS containing 3% BSA) for 1 hr and then incubated with a mouse anti-Flag antibody (8146S, Cell Signaling, 1:250) diluted in blocking buffer for 2 hr at RT. After four washes, cells were incubated for 1 hr with a goat anti-mouse Alexa Fluor 488-conjugated secondary antibody (Invitrogen) diluted in blocking buffer. Nuclei were stained with mounting medium containing 4',6-diamidino-2-pheylindole (Vector

Laboratories). Images were obtained with a Leica confocal microscope (Leica Biosystems) using an oil immersion objective and excitation wavelengths of 480 and 510 nm.

To analyze Rad51 and γH2AX focus formation, IARS1-depleted U2OS cells were transferred to LabTek™ 4-well chamber slides at a density of 7×10^4 cells per well. They were exposed to ionic radiation using a RS 2000 irradiator (Rad Source) at a dose rate of 10 Gy min^{-1}. Cells were pre-extracted with CSK buffer (10 mM PIPES, 100 mM NaCl, 300 mM sucrose, 3 mM $MgCl_2$, 1 mM EGTA, and 0.5% Triton X-100) for 10 min on ice and fixed with 4% paraformaldehyde for 20 min at RT. After blocking for 30 min at RT, cells were incubated with the indicated antibodies diluted in blocking buffer overnight at 4°C. Rabbit anti-Rad51 (8875S, Cell Signaling, 1:1,000) and goat anti-γH2AX (05-636, Millipore, 1:1,000) antibodies were used. After three washes with PBS containing 0.05% Triton X-100, cells were incubated with an Alexa Fluor 488-conjugated secondary antibody for 30 min and mounted using ProLong® Gold antifade reagent (Vector Laboratories). Confocal images were acquired with an LSM880 confocal microscope (Carl Zeiss). Image acquisition and analysis were performed with ZEN blue software (Carl Zeiss).

Laser microirradiation

A total of 3×10^5 U2OS cells were plated in confocal dishes (SPL), incubated for 1 day, and transfected with 2 μg of a GFP-EXO1-expressing plasmid using Lipofectamine 3000 according to the manufacturer's instructions. After 4 hr, media were replaced by media containing 10 μM 5-bromo-2'-deoxyuridine. The following day, laser microirradiation was performed using a 355 nm ultraviolet A laser, and cells were incubated in a 37°C chamber containing 5% CO_2. After each laser microirradiation, images of cells were obtained every 10 sec for 5 min using a LSM880 confocal microscope. The intensity of each laser strip was determined using Zen blue software, and the values were normalized against the baseline values. At least ten cells were used for quantification.

Supplementary Table 1 Data collection and refinement statistics for *Gg*EARS1–IARS1 and *Gg*EARS1 crystals.

	*Gg*EARS1–IARS1 (PDB 7WRS) XFEL	*Gg*EARS1 (PDB 7WRU) Synchrotron
**Data collection**		
Space group	$P2_12_12$	$P2_1$
Cell dimensions		
a, *b*, *c* (Å)	143.0, 155.5, 44.2	89.0, 41.8, 92.2
α, β, γ (°)	90, 90, 90	90, 100, 90
Wavelength	1.2782	1.0094
Resolution (Å)	77.8-2.4 (2.44-2.4)*	45.4-2.5 (2.54-2.5)*
R_{split}	14.38 (64.9)	
R_{merge}		0.355 (2.656)
$CC_{1/2}$	0.995 (0.866)	0.976 (0.612)
$I / \sigma I$	5.2 (1.8)	8.1 (1.2)
Completeness (%)	100.0 (100.0)	98.4 (99.2)
Redundancy	622.9 (431.1)	6.5 (6.5)
**Refinement**		
Resolution (Å)	71.5-2.4	45.4-2.5
No. reflections	39,660	23,252
R_{work} / R_{free}	19.3 / 24.3	23.2 / 27.4
No. atoms	6384	4005
Protein	6358	4000
Ligand/ion	0	2
Water	26	3
B-factors		
Protein	57.0	54.5
Ligand/ion		162.2
Water	43.7	37.7
R.m.s. deviations		
Bond lengths (Å)	0.008	0.012
Bond angles (°)	0.957	1.14

Number of crystals used for XFEL *Gg*EARS1–IARS1 structure – 2×10^7, for *Gg*EARS1 – a single.
*Values in parentheses are for highest-resolution shell.

Supplementary Table 2 Sequence of qPCR primers and probes used in this study.

BRCA1	Primer FW	TCAAACTTGCATGTGGAGCC
	Primer REV	ACACAGGGGATCAGCATTCA
β-actin	Primer FW	ACACCCCAGCCATGTACGTAGCC
	Primer REV	AAGAGCCTCAGGGCAACGGAACC
DSB1-335 bp	Primer FW	GAATCGGATGTATGCGACTGATC
	Primer REV	TTCCAAAGTTATTCCAACCCGAT
	Probe	6FAM-CACAGCTTGCCCATCCTTGCAAACC-TAMRA
DSB1-1618 bp	Primer FW	TGAGGAGGTGACATTAGAACTCAGA
	Primer REV	AGGACTCACTTACACGGCCTTT
	Probe	6FAM-TTGCAAGGCTGCTTCCTTACCATTCAA-TAMRA
DSB1-3500 bp	Primer FW	TCCTAGCCAGATAATAATAGCTATACAAACA
	Primer REV	TGAATAGACAGACAACAGATAAATGAGACA
	Probe	6FAM-ACCCTGATCAGCCTTTCCATGGGTTAAG-TAMRA
No DSB	Primer FW	ATTGGGTATCTGCGTCTAGTGAGG
	Primer REV	GACTCAATTACATCCCTGCAGCT
	Probe	6FAM-TCTCTGCACAGACCGGCTTCCCTTC-TAMRA

Supplementary References

1. Nureki, O. et al. Structure of an archaeal non-discriminating glutamyl-tRNA synthetase: a missing link in the evolution of Gln-tRNAGln formation. *Nucleic Acids Res.* **38**, 7286–7297 (2010).

2. Fu, Y. et al. Structure of the ArgRS-GlnRS-AIMP1 complex and its implications for mammalian translation. *Proc. Natl. Acad. Sci. USA* **111**, 15084–15089 (2014).

3. Rath, V. L. et al. How glutaminyl-tRNA synthetase selects glutamine. *Structure* 6, 439–449 (1998).

4. Menges, C.W. et al. FAS-associated factor 1 (FAF1): diverse functions and implications for oncogenesis. *Cell Cycle* **15,** 2528-2534 (2009).
5. Otwinowski, Z. and Minor, W. Processing of X-ray diffraction data collected in oscillation mode. *Methods Enzymol.* **276**, 307–326 (1997).
6. McCoy, A. J. et al. Phaser crystallographic software. *J. Appl. Crystallogr.* **40**, 658–674 (2007).
7. Adams, P.D. et al. PHENIX: a comprehensive Python-based system for macromolecular structure solution. *Acta Crystallogr. D Biol. Crystallogr.* **66**, 213–221 (2010).
8. Emsley, P. & Cowtan, K. Coot: model-building tools for molecular graphics. *Acta Crystallogr. D Biol. Crystallogr.* **60**, 2126–2132 (2004).
9. Park, J. et al. Current status of the CXI beamline at the PAL-XFEL. *J. Korean Phys. Soc.* **69**,1089–1093 (2016).
10. Kang, H.S. et al. Hard X-ray free-electron laser with femtosecond-scale timing jitter. *Nat. Photonics* **11**, 708–713 (2017).
11. Kim, J. et al. Focusing X-ray free-electron laser pulses using Kirkpatrick-Baez mirrors at the NCI hutch of the PAL-XFEL. *J. Synchrotron Radiat.* **25**, 289–292 (2018).
12. Mariani, V. et al. *OnDA*: online data analysis and feedback for serial X-ray imaging. *J. Appl. Crystallogr.* **49**, 1073–1080 (2016).
13. Barty, A. et al. *Cheetah*: software for high-throughput reduction and analysis of serial femtosecond X-ray diffraction data. *J. Appl. Crystallogr.* **47**, 1118–1131 (2014).
14. White, T.A. et al. CrystFEL: a software suite for snapshot serial crystallography. *J. Appl. Crystallogr.* **45**, 335–341 (2012).
15. Battye, T.G.G. et al. IMosflm: a new graphical interface for diffraction-image processing with MOSFLM. *Acta Cryst.* **D67,** 271-281 (2011).
16. Livak, K.J. and Schmittgen, T.D. Analysis of relative gene expression data using real-time quantitative PCR and the 2(-Delta Delta C(T)) Method. *Methods* **25,** 402-408 (2001).
