# Supplementary material for: Regulation of BRCA1 stability through the tandem UBX domains of isoleucyl-tRNA synthetase 1
Source: Nat Commun. 2022 Nov 8;13:6732. doi: 10.1038/s41467-022-34612-y (PMC9643514; doi:10.1038/s41467-022-34612-y)
Supplement: Supplementary file 3 — Reporting Summary [file 41467_2022_34612_MOESM3_ESM.pdf]

nature portfolio

Corresponding author(s): Kyungjae Myung, Yunje Cho, NCOMMS-22-07550A

Last updated by author(s): Oct 26, 2022

Reporting Summary

Nature Portfolio wishes to improve the reproducibility of the work that we publish. This form provides structure for consistency and transparency in reporting. For further information on Nature Portfolio policies, see our Editorial Policies and the Editorial Policy Checklist.

Statistics

For all statistical analyses, confirm that the following items are present in the figure legend, table legend, main text, or Methods section.

n/a	Confirmed	
☐	☒	The exact sample size (*n*) for each experimental group/condition, given as a discrete number and unit of measurement
☐	☒	A statement on whether measurements were taken from distinct samples or whether the same sample was measured repeatedly
☐	☒	The statistical test(s) used AND whether they are one- or two-sided<br>*Only common tests should be described solely by name; describe more complex techniques in the Methods section.*
☒	☐	A description of all covariates tested
☒	☐	A description of any assumptions or corrections, such as tests of normality and adjustment for multiple comparisons
☐	☒	A full description of the statistical parameters including central tendency (e.g. means) or other basic estimates (e.g. regression coefficient) AND variation (e.g. standard deviation) or associated estimates of uncertainty (e.g. confidence intervals)
☐	☒	For null hypothesis testing, the test statistic (e.g. *F*, *t*, *r*) with confidence intervals, effect sizes, degrees of freedom and *P* value noted<br>*Give P values as exact values whenever suitable.*
☒	☐	For Bayesian analysis, information on the choice of priors and Markov chain Monte Carlo settings
☒	☐	For hierarchical and complex designs, identification of the appropriate level for tests and full reporting of outcomes
☒	☐	Estimates of effect sizes (e.g. Cohen's *d*, Pearson's *r*), indicating how they were calculated

Our web collection on statistics for biologists contains articles on many of the points above.

Software and code

Policy information about availability of computer code

Data collection	XFEL data were collected at the SFX instrument at PAL-XFEL NCI using OnDA v.2016 (https://journals.iucr.org/j/issues/2016/03/00/zf5001) and pre-processed using Cheetah v. 2019-1.<br>Synchrotorn data collection was performed at PAL beamlines 5C and 11C. All programs typically used at these beamlines were used.
Data analysis	The following software was used in in X-ray diffraction data processing and model building: HKL2000, Cheetah v.2017.3, CrystFEL v.0.6.2, MOSFLM v.7.2.1, Coot v. 0.8.9.1, PyMOL v. 2.3.0, Phaser v.2.1, and Phenix v. 1.14.<br>The cell-based assays data were analysed by GraphPad Prism 8.0. For LC-MS/MS analysis, raw data were analyzed using Scaffold Software v4.0. Proteomics data were analysed by Proteome Discoverer Sorcerer v. 2.1.<br>For immunofluorescence staining, the raw data sets were analyzed using Zen imaging software v7.0.

For manuscripts utilizing custom algorithms or software that are central to the research but not yet described in published literature, software must be made available to editors and reviewers. We strongly encourage code deposition in a community repository (e.g. GitHub). See the Nature Portfolio guidelines for submitting code & software for further information.

Data

Policy information about availability of data

All manuscripts must include a data availability statement. This statement should provide the following information, where applicable:

- Accession codes, unique identifiers, or web links for publicly available datasets
- A description of any restrictions on data availability
- For clinical datasets or third party data, please ensure that the statement adheres to our policy

Atomic coordinates and the structure factors have been deposited in the RCSB under the following accession numbers: PDB 7WRS [https://doi.org/10.2210/

pdb7WRS/pdb] (EARS1-IARS1 complex) and 7WRU [https://doi.org/10.2210/pdb7WRU/pdb] (apo EARS1). The mass spectrometry proteomics data have been deposited to the ProteomeXchange Consortium via the PRIDE partner repository with the dataset identifiers PXD031261 and 10.6019/PXD031261.

Field-specific reporting

Please select the one below that is the best fit for your research. If you are not sure, read the appropriate sections before making your selection.

☒ Life sciences ☐ Behavioural & social sciences ☐ Ecological, evolutionary & environmental sciences

For a reference copy of the document with all sections, see nature.com/documents/nr-reporting-summary-flat.pdf

Life sciences study design

All studies must disclose on these points even when the disclosure is negative.

Sample size	Approximately 20 millions of protein complex (EARS1-IARS1) crystals used to collected data using a fixed-target method at PAL-XFEL and data were integrated and scaled to ensure 100% completeness (431-fold multiplicity at highest resolution shell) of the dataset. Single crystals (apo EARS1) were used for synchrotron diffraction data collection of the protein crystals. The sample size for each experiment group is detailed in the Figure Legends and Material and Methods section.
Data exclusions	Failed immunoblots that could not be quantified because of low signal to noise ratios were excluded.
Replication	Each experiment involved n=3 independent experiments. For quantitative measurements, three or more independent experiments were carried out and statistical analysis performed. All attempts at replication were successful.
Randomization	For cell-based experiments Western blotting, cell types were known when prepare the samples or start to treat cells at the beginning of experiments. Randomization was performed on capturing regions of interests (ROIs) in fluorescence image analyses. In other experiments, rigorous and unbiased quantifications were performed using analyzer (ImageJ and Image Lab).
Blinding	Blinding was not required for this study because no subjective allocation was involved.

Reporting for specific materials, systems and methods

We require information from authors about some types of materials, experimental systems and methods used in many studies. Here, indicate whether each material, system or method listed is relevant to your study. If you are not sure if a list item applies to your research, read the appropriate section before selecting a response.

Materials & experimental systems

n/a	Involved in the study	
☐	☒	Antibodies
☐	☒	Eukaryotic cell lines
☒	☐	Palaeontology and archaeology
☒	☐	Animals and other organisms
☒	☐	Human research participants
☒	☐	Clinical data
☒	☐	Dual use research of concern

Methods

n/a	Involved in the study	
☒	☐	ChIP-seq
☐	☒	Flow cytometry
☒	☐	MRI-based neuroimaging

Antibodies

Antibodies used

Antibodies were obtained from the following sources:
Mouse monoclonal anti-Flag (WB dilution 1:2000, Sigma-Aldrich, F1804, RRID: AB_262044)
Mouse monoclonal anti-GAPDH (0411) (WB dilution 1:5000, Santa cruz, sc-47724, RRID: AB_627678),
Mouse monoclonal anti-Ubiquitin (P4D1) (WB dilution 1:200, Santa cruz, sc-8017, RRID: AB_628423),
Rabbit polyclonal anti-BRCA1 (C-20) (WB dilution 1:250, Santa cruz, sc-642, RRID: AB_630944),
Mouse monoclonal anti-BRCA1 (D-9) (WB dilution 1:250, Santa cruz, sc-6954, RRID: AB_626761),
Mouse monoclonal anti-BARD1 (E-11) (WB dilution 1:500, Santa cruz, sc-74559, RRID: AB_2061237),
Mouse monoclonal anti-IARS1 (D-9) (WB dilution 1:1000, Santa cruz, sc-271826, RRID:AB_10709166),
Mouse monoclonal anti-HA (F-7) (WB dilution 1:1000, Santa cruz, sc-7392, RRID: AB_627809),
Mouse monoclonal anti-Lamin A/C (E-1) (WB dilution 1:1000, Santa cruz, sc-376248, RRID: AB_10991536),
Mouse monoclonal anti-Actin (Clone C4) (WB dilution 1:10,000, MP Biomedicals, 691001, RRID:AB_2335127),
Rabbit polyclonal anti-AIMP3 (WB dilution 1:1000, Abcam, ab155689),
Rabbit monoclonal anti-USP14 (WB dilution 1:2000, Abcam, ab192618),
Rabbit polyclonal anti-EPRS1 (WB dilution 1:2000, Abcam, ab31531, RRID:AB_880047),
Rabbit polyclonal anti-Tubulin (WB dilution 1:2000, Abcam, ab15568, RRID:AB_2210952),
Rabbit polyclonal anti-Lamin B1 (WB dilution 1:1000, Abcam, ab65986, RRID: AB_1140888),
Rabbit polyclonal anti-EXO1 (WB dilution 1:2000, Abcam, ab95012, RRID: AB_10675718),

Mouse monoclonal anti-Myc (Clone 4A6) (WB dilution 1:1000, Millipore, 05-724, RRID: AB_309938),
Mouse monoclonal anti-puromycin (Clone 12D10) (WB dilution 1:1000, Millipore, MABE343, RRID: AB_2566826),
Rabbit monoclonal anti-CDK9 (WB dilution 1:1000, Cell signaling, #2316, RRID: AB_2291505),
Rabbit monoclonal anti-CtIP (D76F7) (WB dilution 1:1000, Cell signaling, #9201, RRID: AB_10828593),
Rabbit polyclonal anti-VDAC (WB dilution 1:1000, Cell signaling, #4866, RRID: AB_2272627),
Mouse monoclonal anti-Flag (9A3) (WB dilution 1:2000, IF dilution 1:200, Cell signaling, #8146, RRID: AB_10950495),
Rabbit polyclonal anti-CDK4 (WB dilution 1:2000, Bethyl laboratories, A304-225A, RRID: AB_2620422),
Rabbit polyclonal anti-LARS1 (WB dilution 1:1000, Bethyl laboratories, A304-315A, RRID: AB_2620511),
IRDye 800CW Donkey polyclonal anti-Mouse IgG secondary (WB dilution 1:10,000, Li-COR, 926-32212, RRID AB_2716622),
Goat polycloanl anti-mouse IgG-HRP secondary antibody (WB dilution 1:5000, Santa cruz, sc-2005, RRID: AB_631736),
Goat polyclonal anti-rabbit IgG-HRP secondary antibody (WB dilution 1:5000, Santa cruz, sc-2004, RRID:AB_631746).

Validation

The antibodies have been commercially obtained and were validated in multiple previous studies. The followings are Research Resource Identifiers (RRIDs) from Resource Identification Portal.
Mouse monoclonal anti-Flag (RRID: AB_262044, https://www.sigmaaldrich.com/KR/ko/product/sigma/f1804),
Mouse monoclonal anti-GAPDH (0411) (RRID: AB_627678, https://www.scbt.com/p/gapdh-antibody-0411),
Mouse monoclonal anti-Ubiquitin (P4D1) (RRID: AB_628423, https://www.scbt.com/p/ubiquitin-antibody-p4d1),
Rabbit polyclonal anti-BRCA1 (C-20) antibody (RRID: AB_630944, https://www.scbt.com/p/brca1-antibody-c-20),
Mouse monoclonal anti-BRCA1 (D-9) (RRID:AB_626761, https://www.scbt.com/p/brca1-antibody-d-9),
Mouse monoclonal anti-BARD1 (E-11) (RRID:AB_2061237, https://www.scbt.com/p/bard1-antibody-e-11),
Mouse monoclonal anti-IARS1 (D-9) (RRID:AB_10709166, https://www.scbt.com/p/ilers-antibody-d-9),
Mouse monoclonal anti-HA (F-7) (RRID:AB_627809, https://www.scbt.com/p/ha-probe-antibody-f-7),
Mouse monoclonal anti-Lamin A/C (E-1) (RRID: AB_10991536, https://www.scbt.com/p/lamin-a-c-antibody-e-1),
Mouse monoclonal anti-Actin (Clone C4) (RRID:AB_2335127, https://www.mpbio.com/us/anti-actin-mouse-monoclonal-antibody-clone-c4),
Rabbit polyclonal anti-AIMP3 (https://www.abcam.com/aimp3p18-antibody-ab155689.html),
Rabbit monoclonal anti-USP14 (https://www.abcam.com/products/primary-antibodies/usp14tgt-antibody-epr15943-c-terminal-ab192618.html),
Rabbit polyclonal anti-EPRS1 (RRID:AB_880047, https://www.abcam.com/glutamyl-prolyl-trna-synthetasepars-antibody-ab31531.html),
Rabbit polyclonal anti-Tubulin (RRID:AB_2210952, https://www.abcam.com/beta-tubulin-antibody-ab15568.html),
Rabbit polyclonal anti-Lamin B1 (RRID: AB_1140888, https://www.abcam.com/lamin-b1-antibody-ab65986.html),
Rabbit polyclonal anti-EXO1 (RRID:AB_10675718, https://www.abcam.com/exonuclease-1-antibody-ab95012.html),
Mouse monoclonal anti-Myc (Clone 4A6) (RRID:AB_309938, https://www.emdmillipore.com/US/en/product/Anti-Myc-Tag-Antibody-clone-4A6,MM_NF-05-724),
Mouse monoclonal anti-puromycin (Clone 12D10) (RRID:AB_2566826, https://www.emdmillipore.com/US/en/product/Anti-Puromycin-Antibody-clone-12D10,MM_NF-MABE343),
Rabbit monoclonal anti-CDK9 (RRID:AB_2291505, https://www.cellsignal.com/products/primary-antibodies/cdk9-c12f7-rabbit-mab/2316),
Rabbit monoclonal anti-CtIP (D76F7) (RRID:AB_10828593, https://www.cellsignal.com/products/primary-antibodies/ctip-d76f7-rabbit-mab/9201),
Rabbit polyclonal anti-VDAC (RRID:AB_2272627, https://www.cellsignal.com/products/primary-antibodies/vdac-antibody/4866),
Mouse monoclonal anti-Flag (9A3) (RRID: AB_10950495, https://www.cellsignal.com/products/primary-antibodies/dykddddk-tag-9a3-mouse-mab-binds-to-same-epitope-as-sigma-s-anti-flag-m2-antibody/8146),
Rabbit polyclonal anti-CDK4 antibody (RRID:AB_2620422, https://www.fishersci.com/shop/products/cdk4-polyclonal-bethyl-laboratories-4/501567174),
Rabbit polyclonal anti-LARS1 (RRID: AB_2620511, https://www.thermofisher.com/antibody/product/LARS-Antibody-Polyclonal/A304-315A),
IRDye 800CW Donkey polyclonal anti-Mouse IgG secondary (RRID AB_2716622, https://www.licor.com/bio/reagents/irdye-800cw-donkey-anti-mouse-igg-secondary-antibody),
Goat polyclonal anti-mouse IgG-HRP secondary antibody (RRID:AB_631736, https://www.scbt.com/p/goat-anti-mouse-igg-hrp),
Goat polyclonal anti-rabbit IgG-HRP secondary antibody (RRID:AB_631746, https://www.scbt.com/p/goat-anti-rabbit-igg-hrp).

Eukaryotic cell lines

Policy information about cell lines

Cell line source(s)

HEK293T, Hela, U2OS, HCT116, insect Sf9 cells were purchased from the American Type Culture Collection (ATCC).

Authentication

The cell lines were authenticated by the supplier (ATCC) using morphology and growth characteristics. Cellular morphology was daily examined.

Mycoplasma contamination

Cell lines were periodically tested for mycoplasma contamination and mycoplasma negative.

Commonly misidentified lines
(See ICLAC register)

No commonly misidentified cell lines were used.

Flow Cytometry

Plots

Confirm that:

☒ The axis labels state the marker and fluorochrome used (e.g. CD4-FITC).

☒ The axis scales are clearly visible. Include numbers along axes only for bottom left plot of group (a 'group' is an analysis of identical markers).

☒ All plots are contour plots with outliers or pseudocolor plots.

☒ A numerical value for number of cells or percentage (with statistics) is provided.

Methodology

Sample preparation	Sample preparations were described in Methods.
Instrument	FACSVerseTM flow cytometer
Software	Data were obtained using BD FACSuiteTM software (BD Biosciences). Data analysis was performed using FlowJo software.
Cell population abundance	Sorting was not performed.
Gating strategy	FSC/SSC gating was used to exclude dead cells and debris.

☐ Tick this box to confirm that a figure exemplifying the gating strategy is provided in the Supplementary Information.
